# Analysis of Pneumocystis Transcription Factor Evolution and Implications for Biology and Lifestyle

**DOI:** 10.1128/mbio.02711-22

**Published:** 2023-01-18

**Authors:** Ryan Ames, Alistair J. P. Brown, Ivana Gudelj, Olga A. Nev

**Affiliations:** a Biosciences and Living Systems Institute, University of Exeter, Exeter, United Kingdom; b Medical Research Council Centre for Medical Mycology, University of Exeter, Exeter, United Kingdom; Duke University

**Keywords:** Pfam, *Pneumocystis*, bioinformatics, fungal pathogens, host-pathogen interactions, phylogenetic analysis, transcription factors

## Abstract

Pneumocystis jirovecii kills hundreds of thousands of immunocompromised patients each year. Yet many aspects of the biology of this obligate pathogen remain obscure because it is not possible to culture the fungus *in vitro* independently of its host. Consequently, our understanding of Pneumocystis pathobiology is heavily reliant upon bioinformatic inferences. We have exploited a powerful combination of genomic and phylogenetic approaches to examine the evolution of transcription factors in Pneumocystis species. We selected protein families (Pfam families) that correspond to transcriptional regulators and used bioinformatic approaches to compare these families in the seven Pneumocystis species that have been sequenced to date with those from other yeasts, other human and plant pathogens, and other obligate parasites. Some Pfam families of transcription factors have undergone significant reduction during their evolution in the Pneumocystis genus, and other Pfam families have been lost or appear to be in the process of being lost. Meanwhile, other transcription factor families have been retained in Pneumocystis species, and some even appear to have undergone expansion. On this basis, Pneumocystis species seem to have retained transcriptional regulators that control chromosome maintenance, ribosomal gene regulation, RNA processing and modification, and respiration. Meanwhile, regulators that promote the assimilation of alternative carbon sources, amino acid, lipid, and sterol biosynthesis, and iron sensing and homeostasis appear to have been lost. Our analyses of transcription factor retention, loss, and gain provide important insights into the biology and lifestyle of Pneumocystis.

## INTRODUCTION

Pneumocystis jirovecii is a major fungal pathogen of humans that infects healthy individuals, often colonizing the lungs of infants in their first year (1, 2). In immunocompromised and transplant patients this fungus causes life-threatening pneumonia (3, 4), and systemic Pneumocystis infections remain among of the most common and serious infections in HIV/AIDS patients (5). The worldwide incidence of Pneumocystis pneumonia exceeds 400,000 cases per year, with mortality rates of 20 to 80%. This is a particularly serious problem for developing countries (such as those in Africa), where populations of HIV-infected individuals are over six times the size of those in developed countries (6). Moreover, Pneumocystis is an emerging problem in non-HIV patients in developed countries such as the United Kingdom (7) and United States (8).

Despite its major impact upon human health, we remain remarkably ignorant about the biology and epidemiology of Pneumocystis. The inability to culture Pneumocystis *in vitro*, despite 3 decades of research, makes this pathogen uniquely difficult to study (9). The lack of *in vitro* culture methods also delayed the generation of full genome sequences, further slowing research on the Pneumocystis genus. Nevertheless, recent genome sequencing has confirmed that the Pneumocystis genus is comprised of several species, each being specific to a particular mammalian host (10). Five Pneumocystis species have been formally described: human-specific Pneumocystis jirovecii (11), mouse-specific Pneumocystis murina (12), rat-specific Pneumocystis carinii (11) and Pneumocystis wakefieldiae (13), and rabbit-specific Pneumocystis oryctolagi (14). Genome sequences for *P. jirovecii* (15, 16), P. murina (15), P. carinii (16, 17), P. wakefieldiae (18), and *P. oryctolagi* (18) have been reported, as have those for the macaque-specific Pneumocystis macacae (18) and dog-specific Pneumocystis canis (18).

The annotation of Pneumocystis genome sequences has revealed that these species possess extremely reduced genomes that lack metabolic genes for pathways that are normally considered essential (16, 19). For example, based on their genomes, Pneumocystis species lack gluconeogenesis and the glyoxylate cycle and are unable to synthesize all 20 essential amino acids as well as some polyamines, lipids, vitamins, and cofactors. Instead, Pneumocystis seems to be dependent on the host for many nutrients, and the fungus probably enjoys a relatively stable environment *in vivo*, as it is predicted to be unable to withstand osmotic, oxidative, and cell wall stresses as well as changes in ambient pH (20). In addition to having evolved these unique host dependencies, Pneumocystis appears to have developed highly efficient strategies to evade the host’s innate and acquired immune defenses, such as potential antigenic variation via major surface glycoprotein (MsG) isoforms and limited expression of the proinflammatory pathogen-associated molecular pattern, β-glucan, by the trophic form (16).

Moreover, genome sequence annotation has suggested that certain families of transcription factors have been significantly reduced during the evolution of Pneumocystis. In particular, in comparison with model fungal species such as Saccharomyces cerevisiae, Schizosaccharomyces pombe, and Candida albicans, the transcription factor families defined by three Pfam domains (fungus-specific transcription factor domain, fungal Zn_2_Cys_6_ binuclear cluster domain, and bZIP transcription factor domain) appear to be remarkably reduced in *P. jirovecii*, P. murina, and P. carinii (16). Indeed, these three Pneumocystis species carry the lowest number of transcription factors with these Pfam domains among the diverse fungal species examined and are comparable only with the microsporidia (16).

Significant insights into fungal pathogenesis have been gained through experimental screens of transcription factor mutants. These have highlighted, for example, the importance of metabolic adaptation, micronutrient assimilation, and stress responses for the establishment of infections by Candida albicans, Cryptococcus neoformans, and Aspergillus fumigatus (21–24). It is not yet possible to culture Pneumocystis *in vitro* (9). Therefore, using bioinformatic approaches, we have explored more deeply the evolution of a range of families of transcriptional regulators in Pneumocystis to gain further insights into the reduced biology of these pathogens. To this end, we selected Pfam families that correspond to transcriptional regulators, rather than components of the transcriptional apparatus *per se*. We then used bioinformatic approaches to compare these families in the seven Pneumocystis species that have been sequenced to date with those from other yeasts, including baker’s yeast (S. cerevisiae), fission yeasts located relatively close to Pneumocystis in the fungal tree of life (S. pombe, Schizosaccharomyces cryophilus, Schizosaccharomyces japonicus, and Schizosaccharomyces octosporus), other human pathogens (C. albicans), a plant pathogen (Taphrina deformans), and other obligate parasites (Encephalitozoon cuniculi and Encephalitozoon intestinalis) whose genomes are also significantly reduced (25, 26). Our investigations of gene retentions, gene losses, and gene gains provide new insight into the host dependence of Pneumocystis species.

## RESULTS

### Rationale.

**FIG 1:**
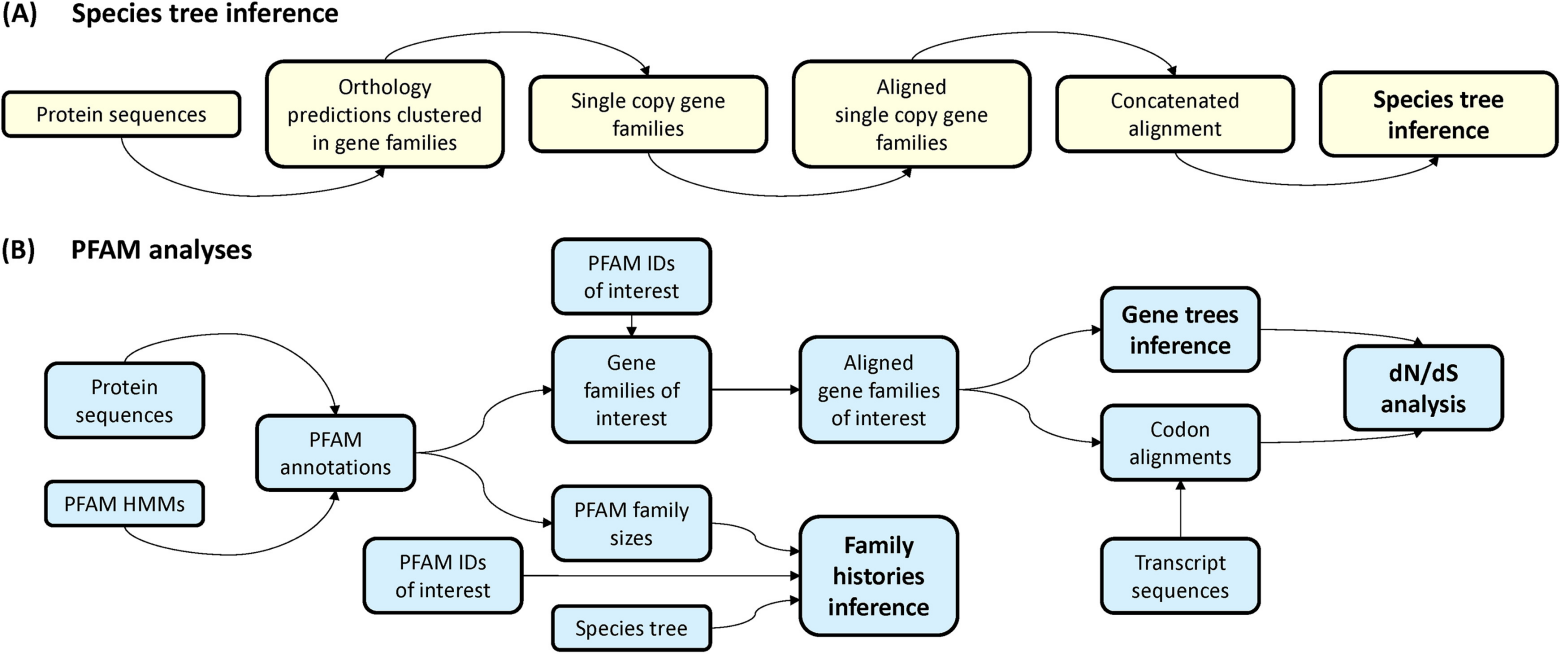
Schematic representation of the bioinformatic approach used in this study. (A) Inference of species trees. (B) Pfam analyses, inference of Pfam family histories in the species examined, and *dN*/*dS* analysis (see the text).

Our aim was to examine the evolution of transcriptional regulators in Pneumocystis species in the context of analogous regulators from benign (S. cerevisiae and S. pombe) and pathogenic (C. albicans) model yeasts, other *Schizosaccharomyces* species that lie relatively close to Pneumocystis in fungal phylogeny (*S. cryophilus*, *S. japonicus*, and S. octosporus), and intracellular parasites that possess reduced genomes (E. cuniculi and E. intestinalis). We screened their annotated sequences for those proteins containing signatures of Pfam families that represent transcriptional regulators (Fig. 1). In particular, we selected Pfam identifiers (IDs) that correspond to regulators that bind directly to DNA and regulatory promoter elements, rather than components of the transcriptional apparatus (Table 1).

**TABLE 1:** Selected PFAM families

Pfam family	Description
PF00170	bZIP transcription factor
PF00172	Fungal Zn_2_Cys_6_ binuclear cluster domain
PF00319	SRF-type transcription factor (DNA-binding and dimerization domain)
PF00320	GATA zinc finger
PF02045	CCAAT-binding transcription factor (CBF-B/NF-YA) subunit B
PF04082	Fungus-specific transcription factor domain
PF05764	YL1 nuclear protein
PF05920	Homeobox KN domain
PF08164	Apoptosis-antagonizing transcription factor, C terminal
PF08731	Transcription factor AFT
PF09011	HMG box domain
PF09341	Transcription factor Pcc1
PF10380	Transcription factor CRF1
PF15227	Zinc finger of C3HC4-type, RING

**FIG 2:**
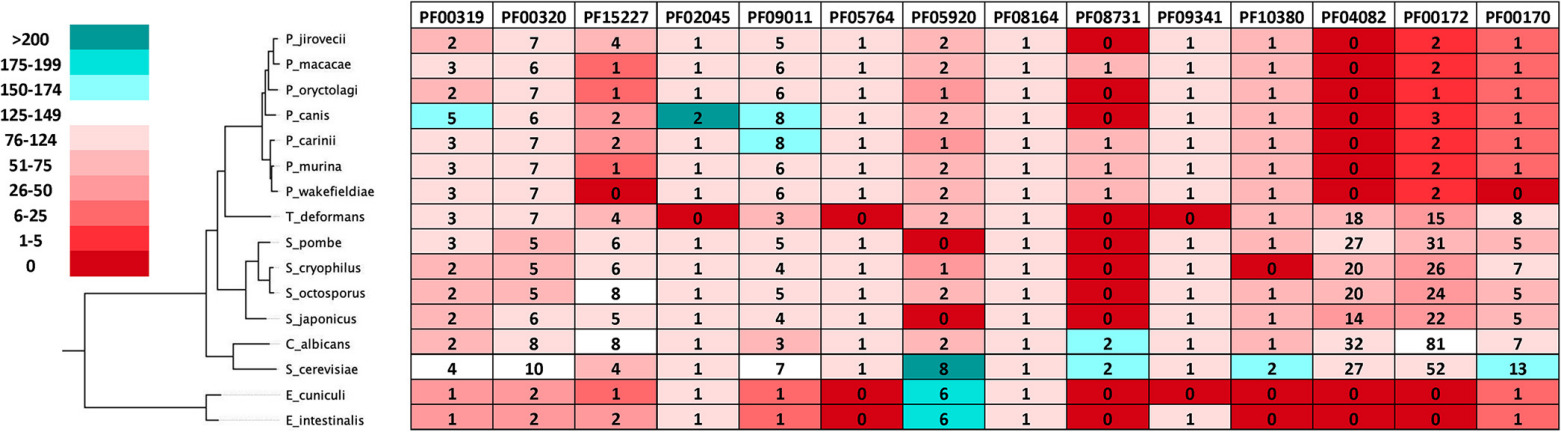
Number of gene members of each Pfam family. The number in each cell represents the number of members for that Pfam family identified in the relevant species. The color reflects the percent change in gene number relative to the average for three model pathogens: S. cerevisiae, S. pombe, and C. albicans (see scale).

In parallel, we reconstructed a species tree for the organisms selected in this study (Fig. 2). This species tree reflects current views of the position of Pneumocystis species relatively close to *Schizosaccharomyces* species in the fungal kingdom (15, 18, 27) and is consistent with current views of the juxtaposition of Pneumocystis species relative to one another (18). For example, the tree places the primate-specific pathogens, *P. jirovecii* and *P. macacae*, close to each other and the rodent-specific pathogens, P. murina and P. carinii, near one another (Fig. 2).

We then compared the number of gene members for each of the Pfam families of interest in the selected species (Fig. 2). As previously reported (16), *P. jirovecii*, P. carinii, and P. murina have significantly fewer members of the Pfam families PF00170, PF00172, and PF04082, which correspond to bZIP, Zn_2_Cys_6_, and fungus-specific transcription factors, respectively, than do other yeasts. Our analyses reveal that like *P. jirovecii*, P. carinii, and P. murina, the other four Pneumocystis species we examined also possess few members of the PF00170 and PF00172 families and have no members of PF04082 (Fig. 2). Furthermore, all the Pneumocystis species have few members of the Pfam families PF05920, PF08731, and PF15227. Interestingly, this pattern of low numbers of transcriptional regulators did not extend to other Pfam families that we examined. For some Pfam families, Pneumocystis species have similar numbers of members as the other fungi examined (PF00320, PF08164, PF10380, PF09341, PF05764, and PF02045), and in some cases, the Pneumocystis species have more members (PF09011 and PF00319). This suggested the selective loss, retention, or gain of specific types of transcriptional regulator during the evolution of Pneumocystis, presumably based on the contribution of these regulators to the lifestyle of these obligate, host-specific pathogens.

To explore this further, we inferred the family history for each selected Pfam ID to predict where these families appear to have lost, retained, or gained genes in the phylogenetic tree of these selected fungal species. We also identified the relative rates of synonymous and nonsynonymous substitutions for each Pfam family (*dN*/*dS* analysis) to gain insight into the degree of selective pressure acting on each protein coding gene and on each Pfam family as a whole (see Materials and Methods).

### Gene retention.

Our analyses revealed that certain transcription factor gene families appear to have been retained in most Pneumocystis species.

In particular, six or seven genes from the GATA sequence binding family PF00320 have been conserved in Pneumocystis species (Fig. 2 and Fig. S5). In S. cerevisiae, members of this Pfam family perform a range of functions, such as the regulation of nitrogen catabolic gene expression (Dal80/YKR034W, Gat1/YFL021W, Gln3/YER040W, and Gzf3/YJL110C), spore wall assembly (Gat3/YLR013W and Gat4/YIR013C), and RNA processing (Ecm23/YPL021W and Srd1/YCR018C). Other fungal species we analyzed have retained 5 to 10 genes from this family.

The Pfam family PF00319 represents SRF-type transcription factors which, in S. cerevisiae, are involved in osmotic stress resistance (Smp1/YBR182C), maintenance of cell integrity (Rlm1/YPL089C), and pheromone and arginine responses (Mcm1/YMR043W and Arg80/YMR042W, respectively). Most Pneumocystis species have retained two or three PF00319 members, while the other fungal species we analyzed have two to four genes in this family (Fig. 2). Compared to the other Pneumocystis species examined, *P. canis* appears to be an outlier, having undergone lineage-specific gains to generate five PF00319 family members. Indeed, *P. canis* seems notable in that, compared to other Pneumocystis species, it has gained additional members in a number of transcriptional regulator families and not lost as many genes in others (Fig. 2 and Fig. S4).

For some Pfam families, most of the fungal species we analyzed, including the Pneumocystis species, have generally retained one or two gene members. These families include PF02045, PF08164, PF05764, PF09341, and PF10380 (Fig. 2).

The PF02045 family contains CCAAT-binding transcription factors. Most of the selected species retain a single gene, with the exception of *T. deformans* (which appears have lost it) and *P. canis* (which has gained an extra family member) (Fig. 2 and Fig. S6). The single PF02045 family member in S. cerevisiae (Hap2/YGL237C) is a global regulator of respiratory gene expression.

**FIG 3:**
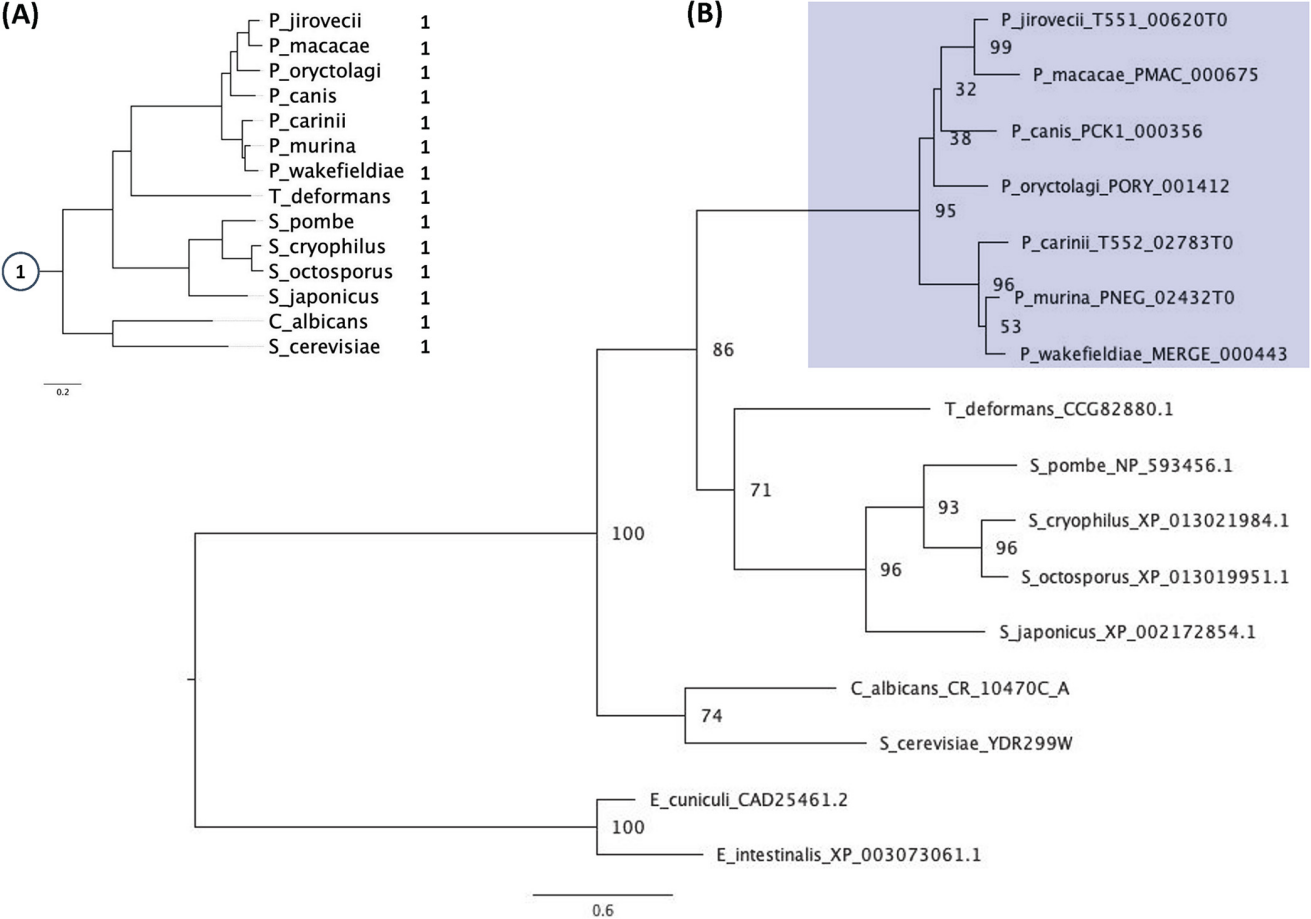
Species and gene phylogeny for the Pfam family PF08164. (A) The species tree shows the evolutionary history of the studied species as well as the predicted ancestral family size at the internal node (in circle) and the number of genes from PF08164 that each species retains. (B) The gene tree (with the bootstrap data displayed on the nodes) shows the predicted evolutionary history of the genes from the PF08164 family for the studied species, highlighting in blue a monophyletic group of genes corresponding to the Pneumocystis clade.

PF08164 (Fig. 3) describes apoptosis-antagonizing transcription factors. Notably, all of the species examined contain a single member of this family (Fig. 2), and in S. cerevisiae, Bfr2/YDR299W is involved in the inhibition of cell death via RNA processing.

With the exception of *T. deformans*, E. cuniculi, and E. intestinalis, which have lost all PF05764 family members, most of the other species examined possess one member of this family (YL1 nuclear protein [Fig. 2 and Fig. S7]). In S. cerevisiae, the sole PF05764 transcription factor, Vps72/YDR485C, is responsible for vacuolar protein sorting.

Almost all of the species examined possess a single member of the PF09341 family (transcription factor PCC1) which, in S. cerevisiae (Pcc1/YKR095W-A), is required for tRNA modification and regulates genes involved in cell cycle progression and polarized growth. Interestingly, there appear to sporadic losses of this family in *T. deformans* and E. cuniculi (Fig. 2 and Fig. S8).

Finally, PF10380 (transcription factor CRF1) falls into this category: the two members of this family in S. cerevisiae (Crf1/YDR223W and Ifh1/YLR223C) regulate ribosomal protein gene transcription. All of the Pneumocystis species carry a single PF10380 member, whereas *S. cryophilus* and both of the microsporidian species examined appear to have lost this Pfam family (Fig. 2 and Fig. S9).

The presence of a homologue in Pneumocystis species does not guarantee that the S. cerevisiae function has been conserved in these pathogens. Nevertheless, taken together, these examples of gene retention suggest that transcriptional regulators involved in key housekeeping functions, such as ribosomal protein gene expression, RNA processing and modification, respiration, inhibition of cell death, and nitrogen catabolism, may have been conserved in Pneumocystis species.

### Gene expansion.

**FIG 4:**
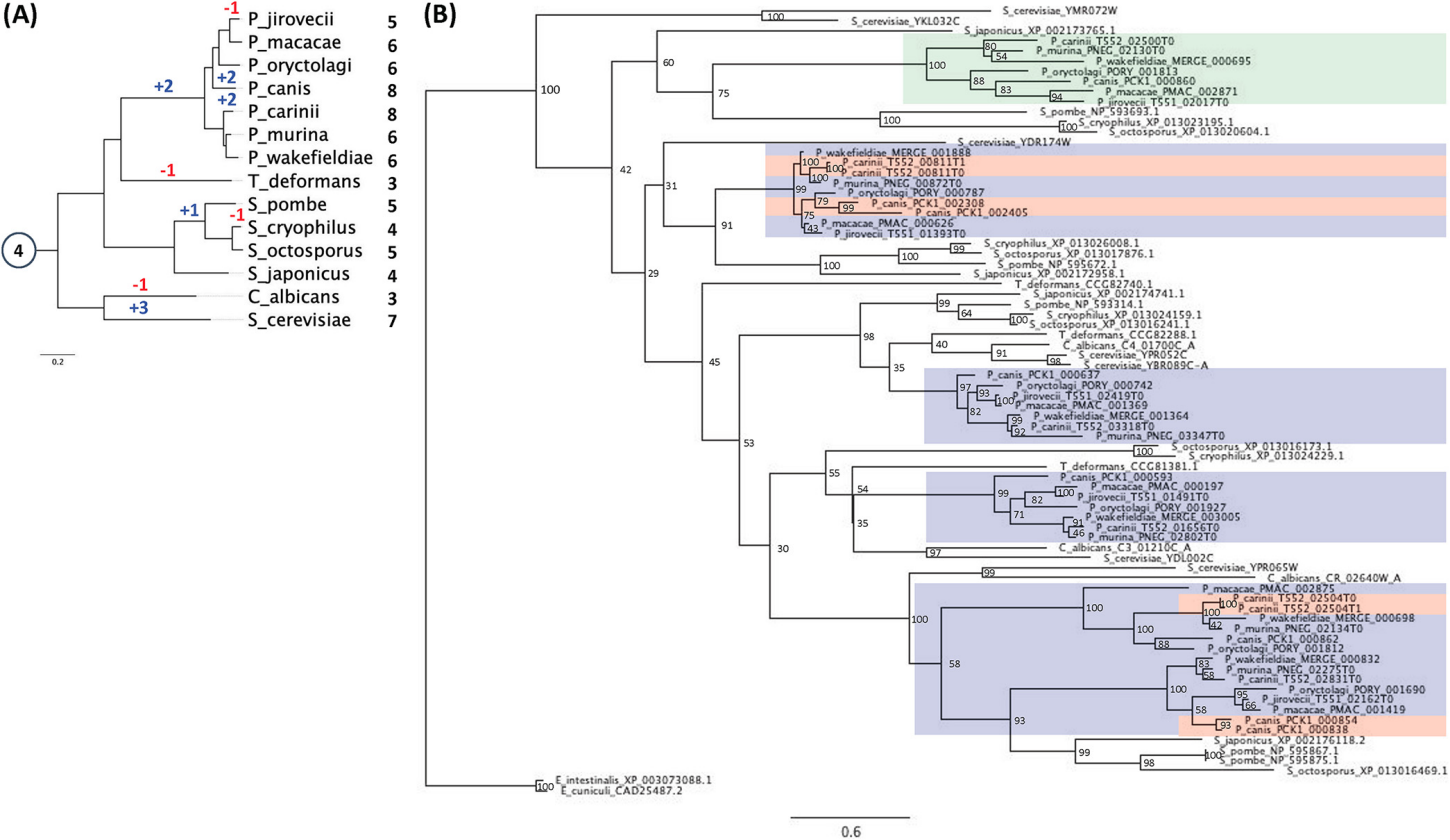
Species and gene phylogeny for the Pfam family PF09011. (A) The species tree shows the evolutionary history of the studied species as well as the ancestral family size at the internal node (in circle) and the number of genes from PF09011 that each species retains. Blue and red numbers along the branches indicate the numbers of gene gains and losses, correspondingly. (B) The gene tree (with the bootstrap data displayed on the nodes) shows the evolutionary history of the genes from the PF09011 family for the studied species, highlighting in blue monophyletic groups of genes corresponding to the Pneumocystis clade. Lineage-specific duplication for certain members of the clade (highlighted in red) as well as gene expansion in Pneumocystis (highlighted in green) have been observed in this Pfam family.

According to our analysis, another Pfam family of transcription factors has undergone expansion in Pneumocystis species (Fig. 2): the HMG box domain family (PF09011). S. cerevisiae has seven family members, but the other non-Pneumocystis species we examined possess one to five members. In contrast to the microsporidia, both of which have a single PF09011 gene, the Pneumocystis species have five to eight family members (Fig. 2). This strongly suggests that functions performed by transcription factors in this Pfam family are important for Pneumocystis. In S. cerevisiae, PF09011 members are involved in chromatin remodeling (Hmo1/YDR174W, Nhp6A/YPR052C, Nhp6B/YBR089C-A, and Nhp10/YDL002C), mitochondrial RNA replication (Abf2/YMR072W), and hypoxic gene regulation (Ixr1/YKL032C and Rox1/YPR065W). Phylogenetic analysis of the PF09011 genes from all the species examined suggests that while homologues of S. cerevisiae Hmo1/YDR174W, Nhp6A/YPR052C, Nhp6B/YBR089C-A, and Nhp10/YDL002C have been conserved, homologues of Rox1/YPR065W have undergone duplication in Pneumocystis species (Fig. 4). This might suggest that hypoxic gene regulation is important for the lifestyle of Pneumocystis. However, it should be noted that in C. albicans, the homologue of Rox1/YPR065W (Rfg1/CR_02640W_A) has been functionally reassigned to regulate yeast-hypha morphogenesis (28).

### Gene reduction.

Given that some transcription factor families have been conserved in Pneumocystis and that another has undergone expansion (see above), it is significant that other transcription factor families have undergone significant genome reduction. Kovacs’s team reported that the Pfam families PF00170 (bZIP transcription factors), PF00172 (Zn_2_Cys_6_ binuclear cluster domain), and PF04082 (fungal specific transcription factor domain) have undergone significant reduction in *P. jirovecii*, P. murina, and P. carinii (16). Here, we extend this analysis to *P. canis*, *P. macacae*, *P. oryctolagi*, and *P. wakefieldiae*, as well as to additional transcription factor families that display significant reduction in this genus.

**FIG 5:**
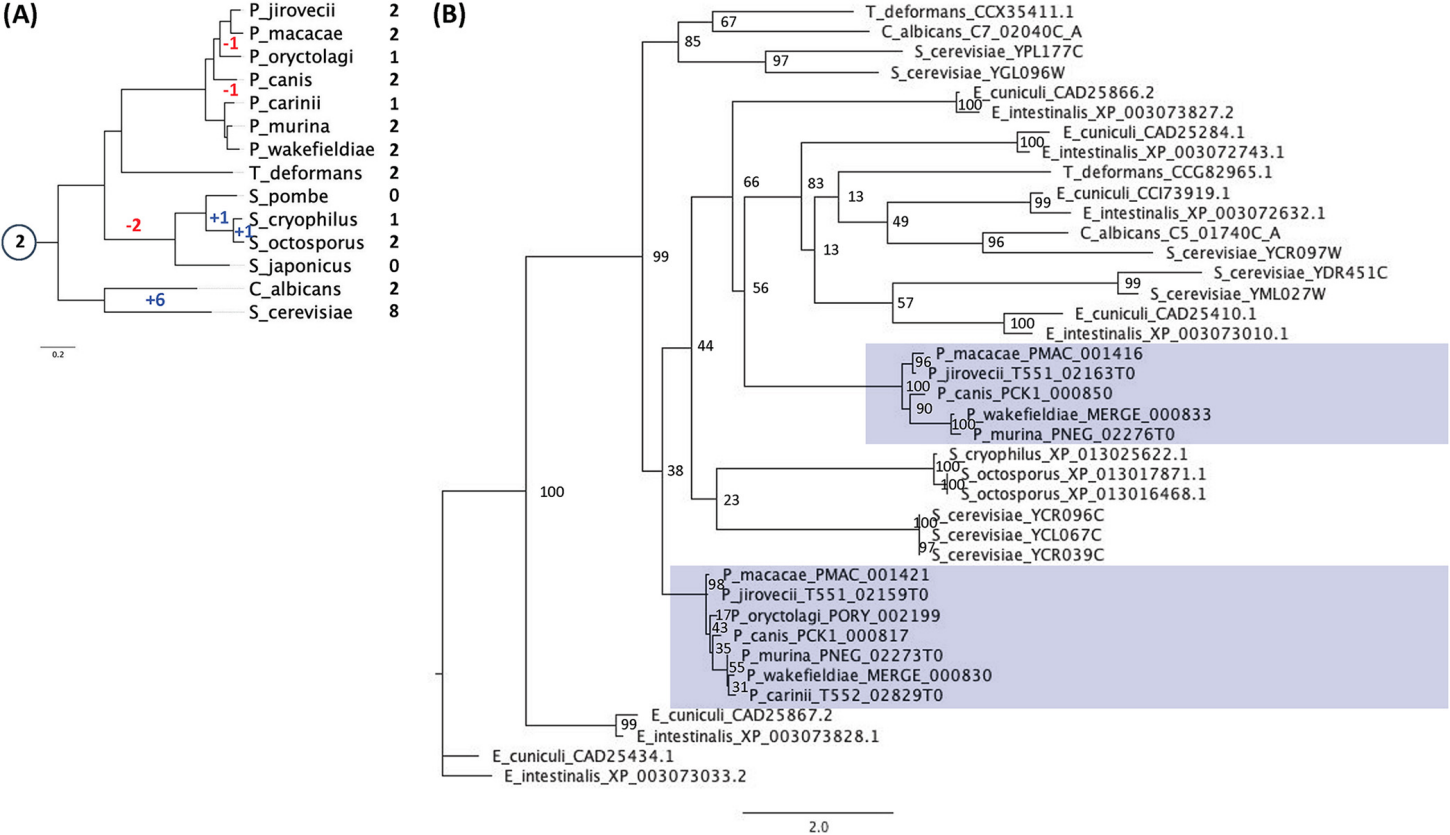
Species and gene phylogeny for the Pfam family PF05920. (A) The species tree shows the evolutionary history of the studied species as well as the ancestral family size at the internal node (in circle) and the number of genes from PF05920 that each species retains. Blue and red numbers along the branches indicate the numbers of gene gains and losses, correspondingly. (B) The gene tree (with the bootstrap data displayed on the nodes) shows the evolutionary history of the genes from the PF05920 family for the studied species, highlighting in blue two monophyletic groups of genes corresponding to the Pneumocystis clade.

The PF05920 family displays considerable variation in gene number across the yeast species examined, but only one or two genes have been retained in the reduced genomes of Pneumocystis species (Fig. 2). This family describes homeobox KN domain proteins, most members of which are involved in the regulation of cell type and cell cycle in S. cerevisiae (Cup9/YPL177C, Hmlα2/YCL067C, HmrA1/YCR097W, HmrA2/YCR096C, Matα2/YCR039C, Tos8/YGL096W, Yhp1/YDR451C, and Yox1/YML027W). Phylogenetic analysis of PF05920 members from all of the yeast species examined clearly shows that the Pneumocystis proteins are clustered into two groups (Fig. 5). These two Pneumocystis clusters lie distant from the S. cerevisiae paralogues Cup9/YPL177C (which regulates the expression of a major peptide transporter) and its paralogue Tos8/YGL096W (which is induced during meiosis and under cell-damaging conditions), suggesting that their functions have proved dispensable in Pneumocystis.

The DupliPHY software, which predicts the most parsimonious number of gains and losses in a gene tree (29), suggested that the PF08731 family might have evolved from an ancestor containing zero family members (see Fig. S1A in the supplemental material). However, this prediction includes several independent instances of *de novo* gene gain during a putative family expansion, which is unlikely. Therefore, we suggest that this gene family is more likely to have evolved from an ancestor that contained at least one PF08731 family member and, consequently, that PF08731 family members have been lost in many of the yeast species analyzed (Fig. 2). For this reason, we suggest that the PF08731 family has actually undergone reduction. The S. cerevisiae PF08731 members (Aft1/YGL071W and Aft2/YPL202C) are AFT transcription factors that are required for intracellular iron homeostasis and oxidative stress resistance. These roles appear to have been conserved in C. albicans (30), but three of the seven Pneumocystis species analyzed have lost this Pfam family (Fig. S1B). Nevertheless, *dN*/*dS* analyses of the single genes remaining in the other four Pneumocystis species suggest that they are under negative selection. Also, their amino acid sequences align well with each other, revealing conservation of aligned domains in blocks of sequence homology (Fig. S1C). Taken together, the gene tree, the *dN*/*dS* analysis, and the amino acid sequence alignments provide evidence of conservation (Fig. S1). Therefore, by inference, the roles of AFT transcription factors in iron homeostasis and/or oxidative stress resistance may have been retained in some Pneumocystis species.

10.1128/mbio.02711-22.1 FIG S1 Species and gene phylogeny for the Pfam family PF08731. (A) The species tree shows the evolutionary history of the studied species as well as the predicted ancestral family size at the internal node (in circle) and the number of genes from PF08731 that each species retains. Blue numbers along the branches indicate the numbers of gene gains. (B) The gene tree (with the bootstrap data displayed on the nodes) shows the predicted evolutionary history of the genes from the PF08731 family for the studied species highlighting in blue a monophyletic group of genes corresponding to the Pneumocystis clade. (C) Protein sequence alignment for the genes and species from the gene tree (B) performed with MUSCLE software (49). Download FIG S1, TIF file, 1.5 MB. Copyright © 2023 Ames et al. 2023 Ames et al. https://creativecommons.org/licenses/by/4.0/ This content is distributed under the terms of the Creative Commons Attribution 4.0 International license.

**FIG 6:**
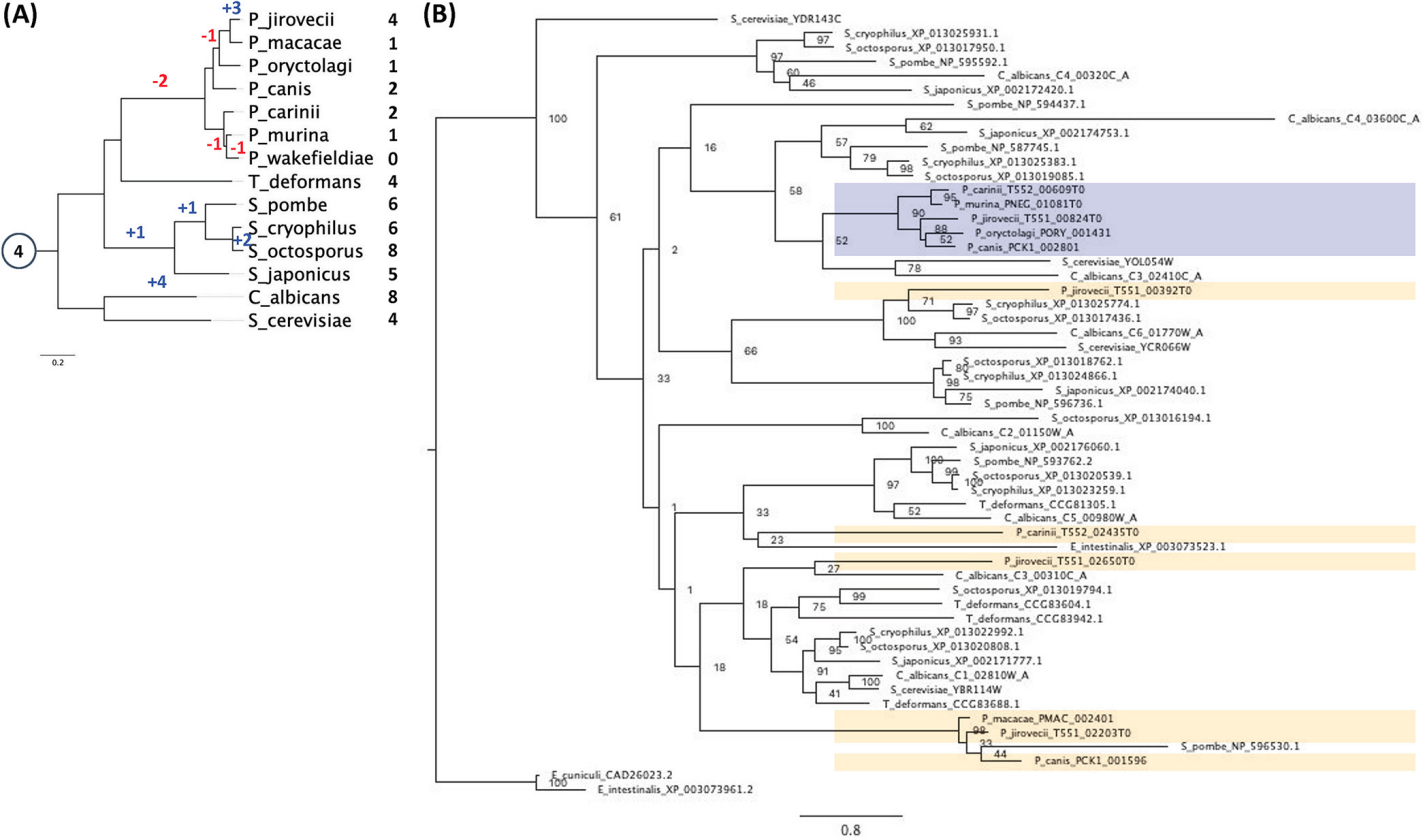
Species and gene phylogeny for the Pfam family PF15277. (A) The species tree shows the evolutionary history of the studied species as well as the ancestral family size at the internal node (in circle) and the number of genes from PF15277 that each species retains. Blue and red numbers along the branches indicate the numbers of gene gains and losses, correspondingly. (B) The gene tree (with the bootstrap data displayed on the nodes) shows the evolutionary history of the genes from the PF15277 family for the studied species highlighting monophyletic (in blue) and paraphyletic (in yellow) groups of genes corresponding to the Pneumocystis clade.

Additional transcription factor families seem to be lost from Pneumocystis species. For example, the free-living yeast species examined contain four to eight members of the PF15227 family (zinc finger of C3HC4-type, RING), whereas the Pneumocystis species possess zero to four members (Fig. 2). Significantly, our phylogenetic analysis of all PF15227 members revealed that with the exception of one cluster which lies close to S. cerevisiae Psh1/YOL054W (an E3 ubiquitin ligase), most Pneumocystis PF15227 proteins do not cluster together (Fig. 6). Therefore, excepting Psh1/YOL054W homologues, PF15227 family members appear to have been differentially lost in different species. This suggests that an ancestral duplication has been followed by (with the exception of Psh1/YOL054W homologues) the differential loss of homologues that are not central to the lifestyle of Pneumocystis.

These findings contrast with the Pfam families PF00170 and PF00172, which Kovacs’s team highlighted in *P. jirovecii*, P. murina, and P. carinii (16). During the dramatic reduction of these families in the course of the evolution of the seven Pneumocystis species we examined (Fig. 2), specific family members have been retained (Fig. S2), suggesting that these transcription factors play important roles in Pneumocystis biology.

10.1128/mbio.02711-22.2 FIG S2 Species and gene phylogeny for the Pfam family PF00170. (A) The species tree shows the evolutionary history of the studied species as well as the predicted ancestral family size at the internal node (in circle) and the number of genes from PF00170 that each species retains. Blue and red numbers along the branches indicate the numbers of gene gains and losses, correspondingly. (B) The gene tree (with the bootstrap data displayed on the nodes) shows the predicted evolutionary history of the genes from the PF00170 family for the studied species, highlighting in blue a monophyletic group of genes corresponding to the Pneumocystis clade. Download FIG S2, TIF file, 2.9 MB. Copyright © 2023 Ames et al. 2023 Ames et al. https://creativecommons.org/licenses/by/4.0/ This content is distributed under the terms of the Creative Commons Attribution 4.0 International license.

In particular, the bZIP transcription factor family (PF00170) has been almost lost in Pneumocystis species, showing a dramatic reduction from 5 to 13 genes in the other yeasts to a maximum of one member in Pneumocystis (Fig. 2). Based on the phylogenetic relationships of PF00170 members, our *dN*/*dS* analyses, and the functions of the S. cerevisiae orthologues in this Pfam family, Pneumocystis species appear to have retained either Yap5/YIR018W (high iron sensing) or its paralogue Yap7/YOL028C (nitric oxide response) (Fig. S2). Meanwhile, Pneumocystis species have lost (or never gained) orthologues of Sko1/YNL167C, Yap1/YML007W, Yap2/YDR423C, Yap3/YHL009C, Yap4/YOR028C, Yap6/YDR259C, Yap8/YPR199C (oxidative, osmotic, and other stress responses), Hac1/YFL031W (unfolded protein response), Aca1/YER045C, Cst6/YIL036W (carbon source utilization), and, significantly, Gcn4/YEL009C (amino acid biosynthesis).

The PF00172 family (Zn_2_Cys_6_ binuclear cluster domain) shows an even more dramatic reduction, from 15 to 81 genes in the other yeasts to only one or three members in Pneumocystis species (Fig. 2). In S. cerevisiae, members of this transcription factor family play important roles in metabolism and stress responses: amino acid metabolism (Arg81/YML099C, Aro80/YDR421W, Cha4/YLR098C, Leu3/YLR451W, Lys14/YDR034C, and Put3/YKL015W); fatty acid, sterol, and lipid metabolism (Asg1/YIL130W, Ecm22/YLR228C, Oaf1/YAL051W, Oaf3/YKR064W, Pip2/YOR363C, Tog1/YER184C, and Upc2/YDR213W); carbon metabolism (Ert1/YBR239C, Gal4/YPL248C, Mal13/YGR288W, Mal33/YBR297W, Rgt1/YKL038W, Sip4/YJL089W, and Znf1/YFL052W); other metabolic pathways (Gsm1/YJL103C, Ppr1/YLR014C, and Thi2/YBR240C); responses to heme and oxygen (Hap1/YLR256W); and drug and stress responses (Asg1/YIL130W, Hal9/YOL089C, Pdr1/YGL013C, Pdr3/YBL005W, Pdr8/YLR266C, Rds1/YCR106W, Rsc3/YDR303C, Rsc30/YHR056C, Stb5/YHR178W, Yrm1/YOR172W, Yrr1/YOR162C, and Znf1/YFL052W). Two monophyletic groups of Pneumocystis genes have been retained within the complex phylogeny of PF00172 family members (Fig. S3). One group is located close to the S. cerevisiae genes Ert1/YBR239C and Gsm1/YJL103C, which play roles in carbon and energy metabolism. The other group does not lie close to S. cerevisiae genes but does lie close to C. albicans Zcf1/C3_02640C_A and Fcr1/C3_06850W_A, which, significantly, are involved in tissue colonization, drug resistance, and filamentation.

10.1128/mbio.02711-22.3 FIG S3 Species and gene phylogeny for the Pfam family PF00172. (A) The species tree shows the evolutionary history of the studied species as well as the predicted ancestral family size at the internal node (in circle) and the number of genes from PF00172 that each species retains. Blue and red numbers along the branches indicate the numbers of gene gains and losses, correspondingly. (B) The gene tree (with the bootstrap data displayed on the nodes) shows the predicted evolutionary history of the genes from the PF00172 family for the studied species, highlighting in blue two monophyletic groups of genes corresponding to the Pneumocystis clade. Download FIG S3, TIF file, 2.4 MB. Copyright © 2023 Ames et al. 2023 Ames et al. https://creativecommons.org/licenses/by/4.0/ This content is distributed under the terms of the Creative Commons Attribution 4.0 International license.

The third transcription factor family highlighted by Kovacs’s team was PF04082 (fungus-specific transcription factor) (16). According to our analysis, this Pfam family is completely lost in all seven of the Pneumocystis species we examined, while the other yeasts possess 18 to 32 members. Members of this family in S. cerevisiae include Asg1/YIL130W, Cat8/YMR280C, Cha4/YLR098C, Dal81/YIR023W, Gal4/YPL248C, Hal9/YOL089C, Hap1/YLR256W, Mal13/YGR288W, Mal33/YBR297W, Pdr1/YGL013C, Pdr3/YBL005W, Pdr8/YLR266C, Ppr1/YLR014C, Put3/YKL015W, Rdr1/YOR380W, Stb5/YHR178W, and Tog1/YER184C. (Note that some of these transcription factors contain more than one Pfam domain and therefore also appear in other families. For example, there is overlap with PF00172 family members.) By inference, based on the roles of these S. cerevisiae transcription factors, Pneumocystis species would appear to have discarded transcriptional regulators that control the assimilation of nonoptimal carbon sources, amino acid catabolism, fatty acid metabolism, pyrimidine biosynthesis processes, heme regulation, and stress and drug responses.

## DISCUSSION

We have explored the evolution of transcriptional regulator families in Pneumocystis by selecting members of specific Pfam categories and comparing these families in Pneumocystis to those in S. cerevisiae, S. pombe, C. albicans, *T. deformans*, and microsporidia. Our aim was to gain new insights into the cellular processes that have been retained by Pneumocystis during its coevolution with the host.

Our study reveals that while some families of transcription factors have undergone significant reduction during the evolution of the Pneumocystis genus, in some cases this has involved the retention of specific family members (e.g., PF00170 and PF00172), whereas in other cases, whole Pfam families have been lost (e.g., PF04082) or appear to be in the process of being lost (e.g., PF08731). Meanwhile, other transcription factor families have been retained in Pneumocystis species (e.g., PF02045, PF00319, PF08164, PF05764, PF09341, and PF10380), and some even appear to have undergone some expansion (PF09011). This is particularly significant in light of the circa 35% reduction in protein-coding genes that has taken place in the Pneumocystis genus (e.g., from 5,863 protein-coding genes in S. cerevisiae to 3,761 in *P. jirovecii* [16]). How can we relate these findings to what is known about Pneumocystis biology?

**FIG 7:**
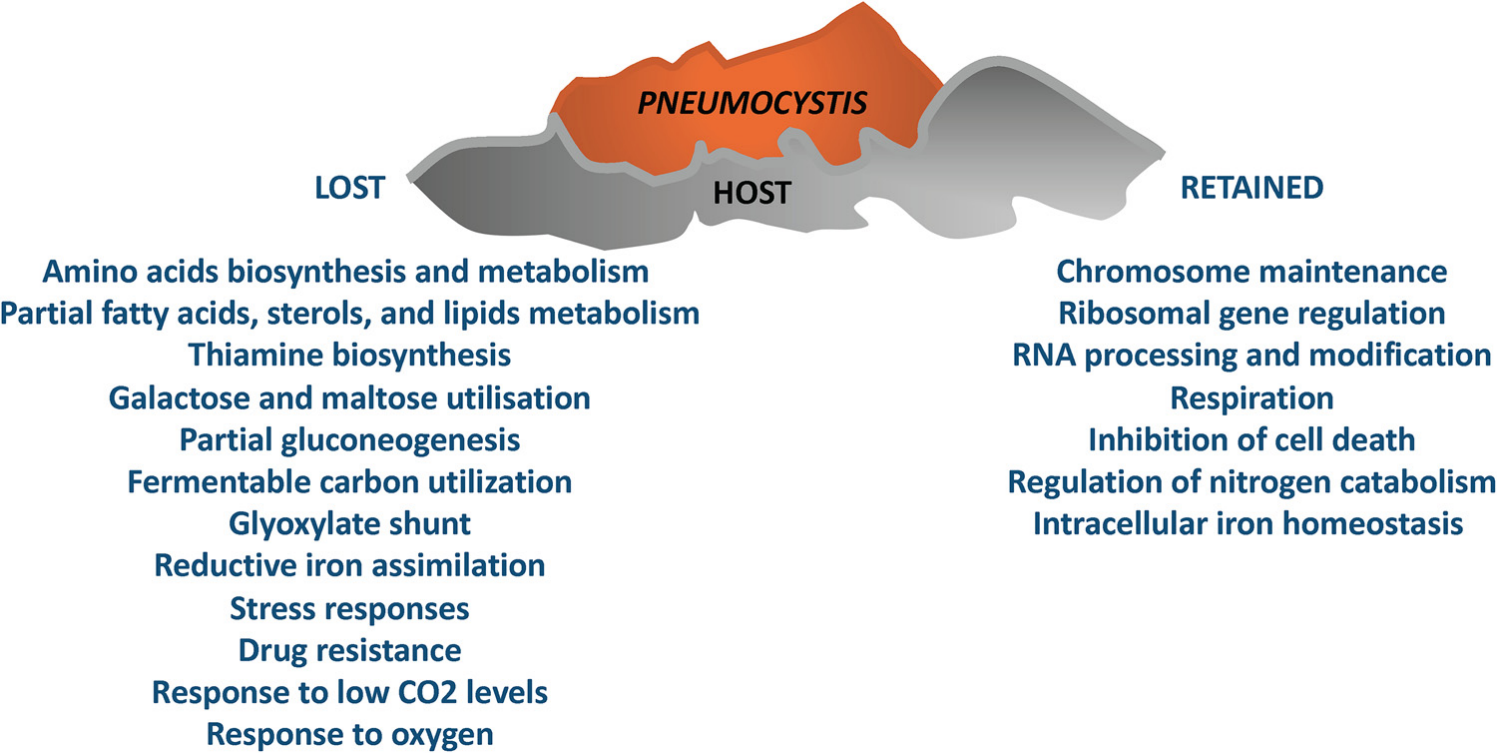
Summary of the major insights into the biology and lifestyle of Pneumocystis: retained and lost biological functions according to the predictions from this study.

Importantly, our results suggest that Pneumocystis species retain essential housekeeping functions, such as chromosome maintenance, ribosomal gene regulation, RNA processing and modification, respiration, and inhibition of cell death as well as regulation of nitrogen catabolism (Fig. 7).

As shown by recent genome sequencing (16, 18), Pneumocystis lacks certain metabolic pathways that are generally essential in free-living yeasts, such as biosynthesis of all 20 essential amino acids as well as some lipids, vitamins, and cofactors. Our results support these bioinformatic inferences by showing that in Pneumocystis species there has been a dramatic reduction in transcription factors related to regulators involved in amino acid synthesis and catabolism in S. cerevisiae (e.g., Arg81/YML099C, Aro80/YDR421W, Cha4/YLR098C, Gcn4/YEL009C, Leu3/YLR451W, Lys14/YDR034C, and Put3/YKL015W). The loss of Gcn4/YEL009C is particularly significant because this global regulator stimulates the synthesis of all amino acids in response to amino acid starvation in evolutionarily divergent fungi (31–33). Orthologues of transcription factors that regulate thiamine biosynthesis and fatty acid, sterol, and lipid metabolism have also been lost from the PF00170 and PF00172 families in Pneumocystis species (Asg1/YIL130W, Ecm22/YLR228C, Oaf1/YAL051W, Pip2/YOR363C, Thi2/YBR240C, Tog1/YER184C, and Upc2/YDR213W). These observations strongly support and extend bioinformatic predictions suggesting that many genes encoding enzymes on these pathways have been lost in *P. jirovecii*, P. murina, and P. carinii (16), indicating that this is also the case for other species in the Pneumocystis genus (Fig. 7).

It has also been predicted that Pneumocystis relies mostly on glycolysis and oxidative pathways for energy production, since genes required for glucose uptake, glycolysis, the tricarboxylic acid cycle, and oxidative phosphorylation, are present in the *P. jirovecii*, P. murina, and P. carinii genomes (20). In addition, the enzymes involved in the conversion of mannose and fructose to glucose seem to be present in Pneumocystis genomes, which infers that these species can also use sugars such as mannose and fructose as alternative carbon sources (20). However, Pneumocystis species lack critical enzymes required for sucrose and galactose assimilation and they are unable to utilize ethanol, acetate, or fatty acids as alternative energy sources, because the genomic data suggest that these species have lost enzymes essential for gluconeogenesis, the glyoxylate cycle, and pyruvate fermentation (20). Our bioinformatic analyses strongly support these inferences, because in addition to losing critical enzymes on these pathways, Pneumocystis species also seem to have lost homologues of key transcription factors that promote the assimilation of alternative carbon sources in S. cerevisiae, such as Cat8/YMR280C, Ert1/YBR239C, Gal4/YPL248C, Mal13/YGR288W, Mal33/YBR297W, Oaf1/YAL051W, Sip4/YJL089W, and Znf1/YFL052W (Fig. 7).

Some regulators of iron sensing and homeostasis also appear to have been lost in Pneumocystis species. This is highly significant, as iron scavenging and homeostasis are critical for the virulence of other fungal pathogens (34–38). According to our analysis, of the eight members of the YAP family in S. cerevisiae (PF00170), only one protein (possibly Yap5/YIR018W or its paralogue Yap7/YOL028C) is retained in six of the seven Pneumocystis species examined (*P. wakefieldiae* appears to have lost all PF00170 family members [Fig. 2]). In S. cerevisiae, the transcription factor Yap5/YIR018W responds to high-iron conditions and regulates vacuolar iron storage in yeast (39, 40). Meanwhile, genomic data suggest that *P. jirovecii*, P. murina, and P. carinii have lost most proteins required for reductive iron assimilation (16). Interestingly, our results indicate that only P. murina, P. carinii, *P. wakefieldiae*, and *P. macacae* have retained an AFT transcription factor (PF08731), whereas other species (*P. jirovecii*, *P. oryctolagi*, and *P. canis*) lack both Aft1/YGL071W and Aft2/YPL202C expressed in S. cerevisiae. Aft1/YGL071W regulates the reductive uptake of iron salts into the cell in S. cerevisiae, and both Aft1/YGL071W and Aft2/YPL202C are required for intracellular iron homeostasis (41). Therefore, Pneumocystis appears to have retained some ability to maintain iron homeostasis in the presence of high iron levels, while discarding the apparatus to control iron scavenging and homeostasis under low-iron conditions (Fig. 7)—properties that are essential for fungal pathogens such as Aspergillus fumigatus, C. albicans, and Cryptococcus neoformans (34–38).

Living in a relatively stable environment in the lung, Pneumocystis appears to have lost many genes involved in responses to osmotic, oxidative, and cell wall stresses as well as to changes in ambient pH (20). This view is strongly supported by our analyses, which clearly show that the Pneumocystis genus has undergone dramatic losses of homologues of transcription factors required for resistance to drugs and environmental stresses. In particular, Pneumocystis species appear to have lost homologues of Sko1/YNL167C (osmotic and oxidative stress), Stb5/YHR178W (multidrug resistance and oxidative stress), Znf1/YFL052W (pH adaptation and osmotic and ethanol stress), Hal9/YOL089C (salt stress), Rsc3/YDR303C and Rsc30/YHR056C (cell wall stress), and Yrr1/YOR162C, Yrm1/YOR172W, Pdr1/YGL013C, Pdr3/YBL005W, and Pdr8/YLR266C (drug resistance). These observations are entirely consistent with the shedding of unnecessary stress responses as Pneumocystis species have undergone evolutionary adaptation to the lung environment (Fig. 7).

Considering the lung environment, the loss of carbonic anhydrase is particularly significant for Pneumocystis (16). This enzyme regulates intracellular pH by catalyzing the interconversion between CO_2_ and bicarbonate and thus providing bicarbonate to cells in fungi (42). Carbonic anhydrase is also critical for the maintenance of the intracellular bicarbonate levels required to maintain adenylyl cyclase activity, and hence protein kinase A signaling, cell growth and morphogenesis (43). However, carbonic anhydrase is no longer essential for C. albicans growth when CO_2_ levels are high (43). Therefore, given that Pneumocystis inhabits the CO_2_-rich environment of the lung, the loss of carbonic anhydrase seems predictable (16). Again, our results support this conclusion because Pneumocystis species lack Cst6/YIL036W homologues (PF00170), a transcription factor required for responses to low CO_2_ levels in S. cerevisiae (44, 45). Moreover, evolutionary adaptation to a relatively stable oxygen supply in the lung environment has led to the loss of the pyruvate fermentation pathway and anaerobic growth genes in Pneumocystis (16). Once again, our analyses indicate that transcription factors involved in regulation of oxygen responses (e.g., Hap1/YLR256W) have been completely lost by Pneumocystis species. Meanwhile, key transcription factors required for respiration (e.g., Hap2/YGL237C) have been retained. This reinforces the importance of aerobic respiration for Pneumocystis species within the lung environment.

We have exploited a powerful combination of genomic and phylogenetic approaches to compare the evolution of transcription factor families in Pneumocystis and other evolutionarily divergent yeasts. This has provided major insights into the biology and lifestyle of Pneumocystis. Given the lack of *in vitro* culture methods for Pneumocystis, this approach has the potential to significantly advance our understanding of this truly unique set of life-threatening fungal pathogens.

## MATERIALS AND METHODS

### Protein and transcript data.

Annotated gene, protein, and transcriptomic sequences for *P. jirovecii*, P. murina, and P. carinii were downloaded from NCBI as deposited by Ma et al. (16). Additional Pneumocystis sequences were taken from Cissé et al. (18), downloading the protein and transcript sequences from the NCBI database. For species with more than one representative strain, we chose *P. macacae* strain P2C and *P. canis* strain CK1—the same strains as the authors used in Fig. 1 of reference 18. There was only a single representative strain for *P. oryctolagi* and *P. wakefieldiae*.

The latest version of protein and transcript sequences for S. cerevisiae (downloaded 2 August 2022) and C. albicans (downloaded 3 October 2022) were downloaded from the *Saccharomyces* Genome Database (46) and *Candida* Genome Database (47), respectively.

For the remaining species included in our analyses, the protein and transcriptomic sequences were downloaded (also 2 August 2022) from the NCBI Genome database for Encephalitozoon cuniculi GB-M1, *Encephalitozoon intestinalis* ATCC 50506, *Schizosaccharomyces cryophilus* OY26, *Schizosaccharomyces japonicus* yFS275, Schizosaccharomyces octosporus (S. octosporus) yFS286, S. pombe, and *T. deformans* PYCC 5710. A summary of the species used in this study, their BioProject accession numbers, and the main characteristics of the assemblies are provided in Table S1.

10.1128/mbio.02711-22.10 TABLE S1 Species and strains used in this study together with their BioProject accession numbers and main characteristics of the genome assemblies. Download Table S1, DOCX file, 0.02 MB. Copyright © 2023 Ames et al. 2023 Ames et al. https://creativecommons.org/licenses/by/4.0/ This content is distributed under the terms of the Creative Commons Attribution 4.0 International license.

### Core and extended analyses.

For some analyses (specified below), it was necessary to remove the *Encephalitozoon* species. To this end, we defined an extended data set (including all species from “Protein and transcript data” above) and a core data set (from which the *Encephalitozoon* species were removed). In the analyses described in “Gene family histories” below, we used a core data set that excluded the *Encephalitozoon* species. This was because the microsporidia, which are distantly related to the fungi in the species tree and have severely reduced genomes, confounded these analyses and reduced our ability to detect gain and loss events in the Pneumocystis lineage.

### Pfam selection.

The following Pfam IDs were selected for analysis: PF00170, PF00172, PF00319, PF00320, PF02045, PF04082, PF05764, PF05920, PF08164, PF08731, PF09011, PF09341, PF10380, and PF15227. Members of these gene/protein families were identified using OMA (Orthologous MAtrix) software version 2.3.0 with default parameters (48): LengthTol: = 0.61; StablePairTol: = 1.81; InparalogTol: = 3.00; ParalogTol: = −2.5*StablePairTol; UseExperimentalHomologousClusters: = false; QuasiCliquesCutoff: = 1.0.

### Species tree inference.

A species tree was inferred using concatenated alignments of 300 single gene copy families identified by OMA in the core data set. Alignments were performed with MUSCLE software version 5.1.1 using default parameters (49). The species tree was inferred using RAxML software version 8.2.12 with the following parameters: random seeds 12345, 100 bootstraps, a full maximum likelihood search for the best scoring tree, and the PROTGAMMAGTR model (50).

### Pfam annotations.

Genes were associated with Pfam IDs and selected for analysis using HMMER3 software version 3.3.2 (51). The Pfam Hidden Markov models (HMMs), Pfam-A.hmm, and descriptions, Pfam-A.clans, were downloaded from the current release Pfam (last update, 15 November 2021). From the HMMER3 software hmmscan was used to search PFAM HMMs against protein sequences for each species in the extended data set. Hits of Pfam domains to sequences were deemed significant if the *i-*value score was no more than 0.00075.

Genes containing significant hits to those Pfam IDs selected for analysis were identified, aligned, and used for phylogenetic (gene) tree inference. In addition, the number of genes with a given Pfam domain in each species (Pfam family size) was counted and used in further gene family analysis (see “Gene family histories” below).

### Gene tree inference.

Gene trees for each selected Pfam family were inferred using a similar approach to that used for inferring the species tree (see “Species tree inference” above). Protein sequences of genes annotated with a target Pfam ID were aligned using MUSCLE software version 5.1.1 with default parameters (49). Phylogenetic (gene) trees were inferred using RAxML software version 8.2.12 with the following parameters: random seeds 12345, 100 bootstraps, a full maximum likelihood search for the best scoring tree, and the PROTGAMMAGTR model (50). This analysis was performed using the extended data set, allowing the *Encephalitozoon* species to act as an outgroup for tree visualization. Gene trees were visualized with FigTree software version 1.4.4 (52).

### Gene family histories.

Gene gains and losses associated with each target Pfam ID of interest were inferred using DupliPHY software, version 1.0 (29). DupliPHY’s weighted parsimony algorithm was run with default parameters to infer the ancestral family sizes at each internal node of the species tree for each selected Pfam ID.

### *dN*/*dS* analysis.

To identify the rates of synonymous and nonsynonymous substitutions for each Pfam family (*dN*/*dS*), protein alignments for each PFAM family were combined with transcript sequences to produce codon alignments. The codon alignments and inferred gene trees for each Pfam family were used to calculate rates of dN/dS for each Pfam family using PAML software version 4.9 with default parameters (53). PAML was run with both the model parameter set to 1 (to estimate a single rate of *dN*/*dS* for the family as a whole) and the model parameter set to 2 (to estimate two or more *dN*/*dS* ratios for branches in the gene phylogeny).

### Data availability.

All data sources are described in the paper.

10.1128/mbio.02711-22.4 FIG S4 Species and gene phylogeny for the Pfam family PF00319. (A) The species tree shows the evolutionary history of the studied species as well as the predicted ancestral family size at the internal node (in circle) and the number of genes from PF00319 that each species retains. Blue and red numbers along the branches indicate the numbers of gene gains and losses, correspondingly. (B) The gene tree (with the bootstrap data displayed on the nodes) shows the predicted evolutionary history of the genes from the PF00319 family for the studied species, highlighting in blue three monophyletic groups of genes corresponding to the Pneumocystis clade. Lineage-specific duplication for certain members of the clade (highlighted in red) have been observed in this Pfam family. Download FIG S4, TIF file, 2.6 MB. Copyright © 2023 Ames et al. 2023 Ames et al. https://creativecommons.org/licenses/by/4.0/ This content is distributed under the terms of the Creative Commons Attribution 4.0 International license.

10.1128/mbio.02711-22.5 FIG S5 Species and gene phylogeny for the Pfam family PF00320. (A) The species tree shows the evolutionary history of the studied species as well as the predicted ancestral family size at the internal node (in circle) and the number of genes from PF00320 that each species retains. Blue and red numbers along the branches indicate the numbers of gene gains and losses, correspondingly. (B) The gene tree (with the bootstrap data displayed on the nodes) shows the predicted evolutionary history of the genes from the PF00320 family for the studied species, highlighting in blue monophyletic groups of genes corresponding to the Pneumocystis clade. Lineage-specific duplication for certain members of the clade (highlighted in red) have been observed in this Pfam family. Download FIG S5, TIF file, 1.0 MB. Copyright © 2023 Ames et al. 2023 Ames et al. https://creativecommons.org/licenses/by/4.0/ This content is distributed under the terms of the Creative Commons Attribution 4.0 International license.

10.1128/mbio.02711-22.6 FIG S6 Species and gene phylogeny for the Pfam family PF02045. (A) The species tree shows the evolutionary history of the studied species as well as the predicted ancestral family size at the internal node (in circle) and the number of genes from PF02045 that each species retains. Blue and red numbers along the branches indicate the numbers of gene gains and losses, correspondingly. (B) The gene tree (with the bootstrap data displayed on the nodes) shows the predicted evolutionary history of the genes from the PF02045 family for the studied species, highlighting in blue a monophyletic group of genes corresponding to the Pneumocystis clade. Lineage-specific duplication for certain members of the clade (highlighted in red) have been observed in this Pfam family. Download FIG S6, TIF file, 2.2 MB. Copyright © 2023 Ames et al. 2023 Ames et al. https://creativecommons.org/licenses/by/4.0/ This content is distributed under the terms of the Creative Commons Attribution 4.0 International license.

10.1128/mbio.02711-22.7 FIG S7 Species and gene phylogeny for the Pfam family PF05764. (A) The species tree shows the evolutionary history of the studied species as well as the predicted ancestral family size at the internal node (in circle) and the number of genes from PF05764 that each species retains. Red numbers along the branches indicate the numbers of gene losses. (B) The gene tree (with the bootstrap data displayed on the nodes) shows the predicted evolutionary history of the genes from the PF05764 family for the studied species, highlighting in blue a monophyletic group of genes corresponding to the Pneumocystis clade. Download FIG S7, TIF file, 2.0 MB. Copyright © 2023 Ames et al. 2023 Ames et al. https://creativecommons.org/licenses/by/4.0/ This content is distributed under the terms of the Creative Commons Attribution 4.0 International license.

10.1128/mbio.02711-22.8 FIG S8 Species and gene phylogeny for the Pfam family PF09341. (A) The species tree shows the evolutionary history of the studied species as well as the predicted ancestral family size at the internal node (in circle) and the number of genes from PF09341 that each species retains. Red numbers along the branches indicate the numbers of gene losses. (B) The gene tree (with the bootstrap data displayed on the nodes) shows the predicted evolutionary history of the genes from the PF09341 family for the studied species, highlighting in blue a monophyletic group of genes corresponding to the Pneumocystis clade. Download FIG S8, TIF file, 2.1 MB. Copyright © 2023 Ames et al. 2023 Ames et al. https://creativecommons.org/licenses/by/4.0/ This content is distributed under the terms of the Creative Commons Attribution 4.0 International license.

10.1128/mbio.02711-22.9 FIG S9 Species and gene phylogeny for the Pfam family PF10380. (A) The species tree shows the evolutionary history of the studied species as well as the predicted ancestral family size at the internal node (in circle) and the number of genes from PF10380 that each species retains. Blue and red numbers along the branches indicate the numbers of gene gains and losses, correspondingly. (B) The gene tree (with the bootstrap data displayed on the nodes) shows the predicted evolutionary history of the genes from the PF10380 family for the studied species, highlighting in blue a monophyletic group of genes corresponding to the Pneumocystis clade. Download FIG S9, TIF file, 1.9 MB. Copyright © 2023 Ames et al. 2023 Ames et al. https://creativecommons.org/licenses/by/4.0/ This content is distributed under the terms of the Creative Commons Attribution 4.0 International license.
