# Supplementary figures and images for: Analysis of Pneumocystis Transcription Factor Evolution and Implications for Biology and Lifestyle
Source: mBio. 2023 Jan 18;14(1):e02711-22. doi: 10.1128/mbio.02711-22 (PMC9973273; doi:10.1128/mbio.02711-22)

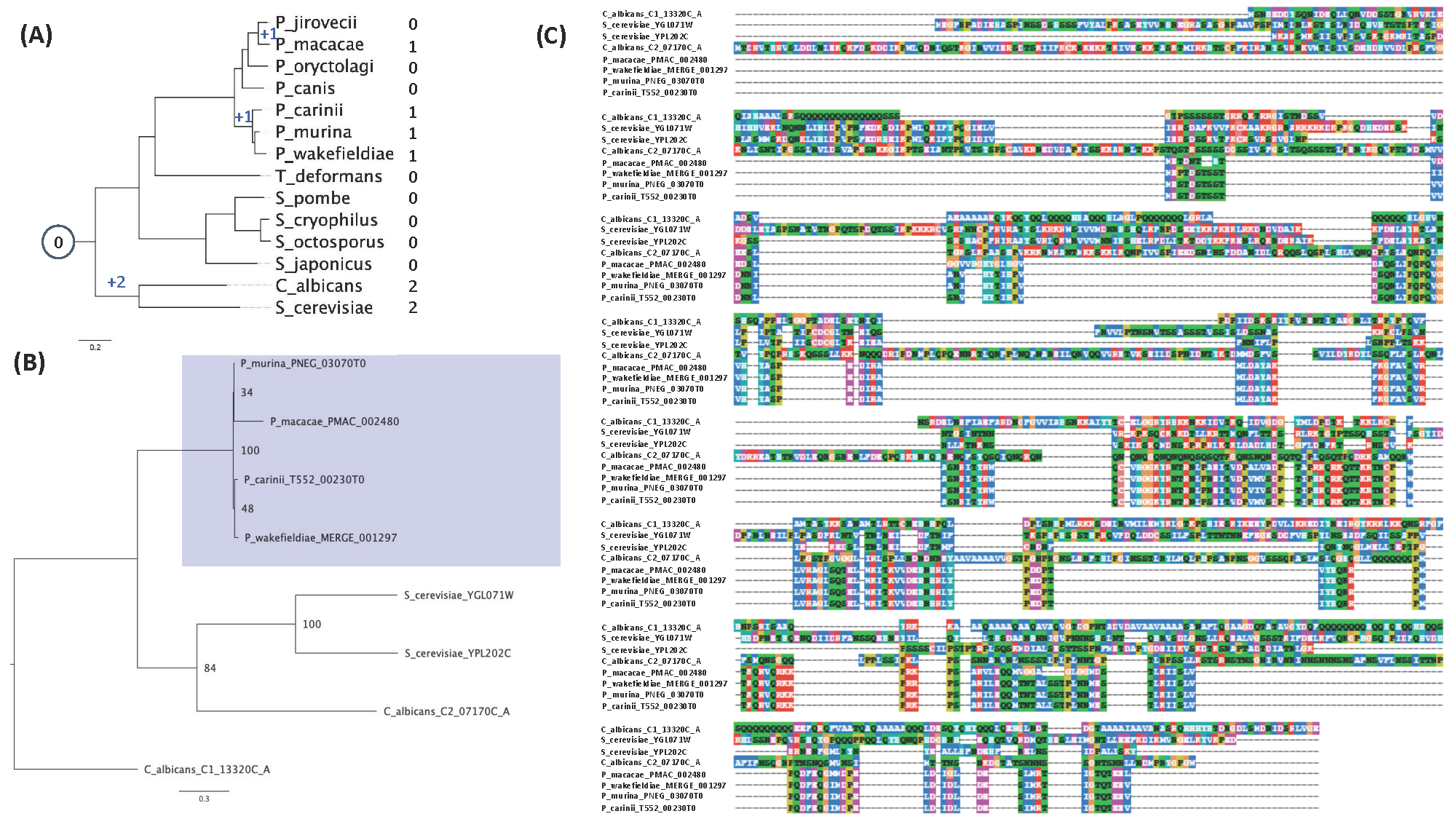

Supplement: FIG S1 [file mbio.02711-22-s0001.tif]

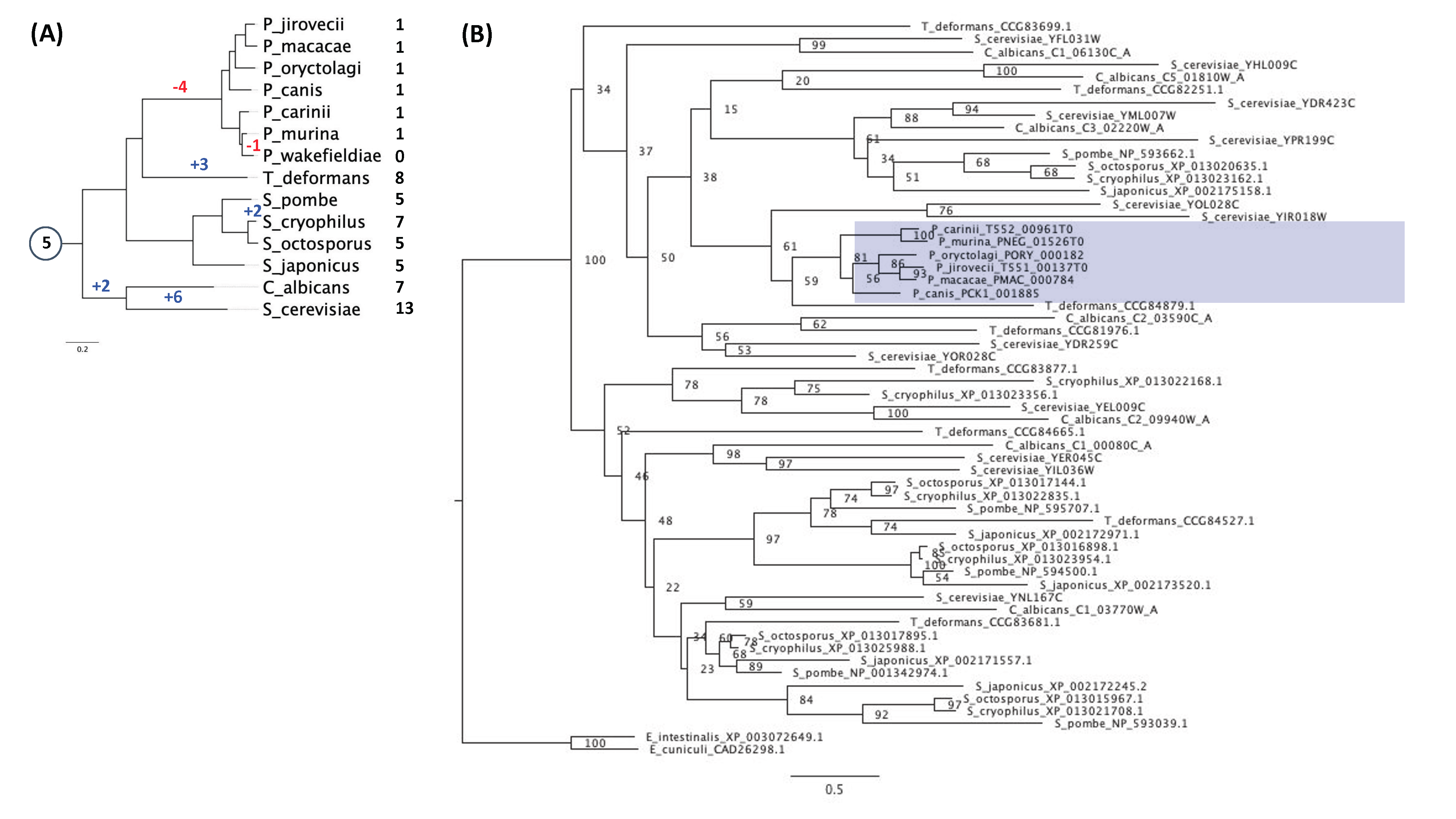

Supplement: FIG S2 [file mbio.02711-22-s0002.tif]

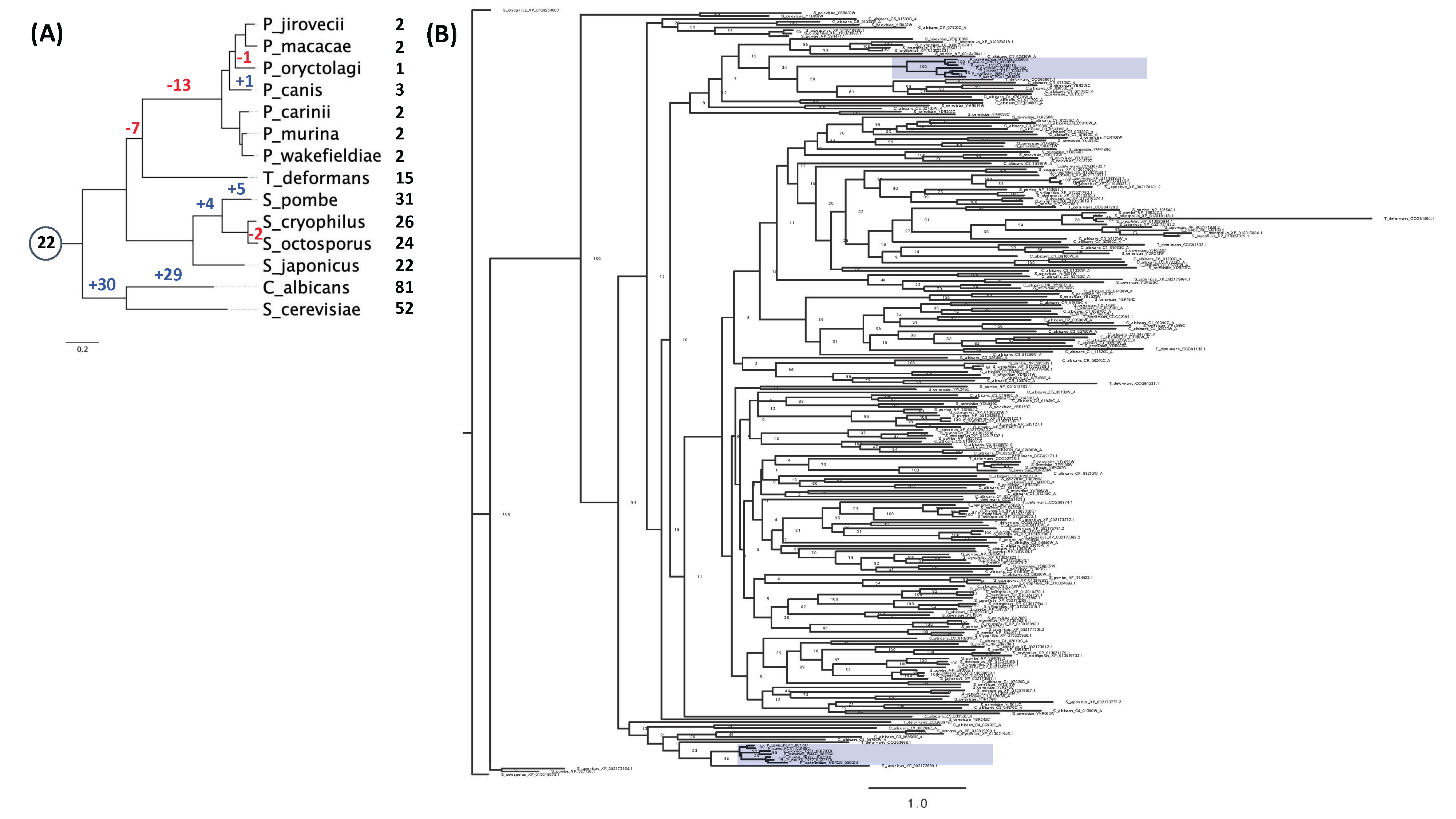

Supplement: FIG S3 [file mbio.02711-22-s0003.tif]

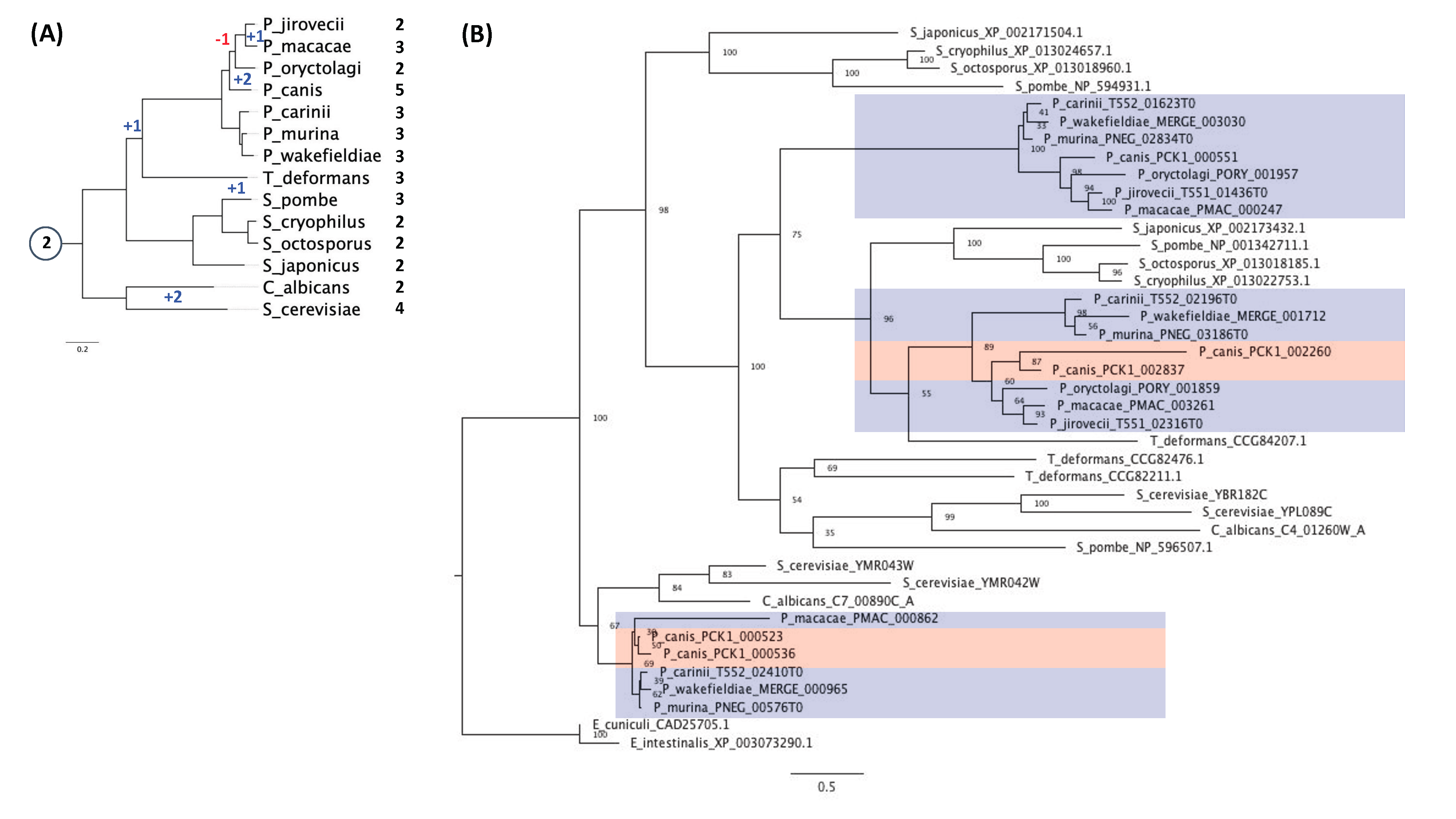

Supplement: FIG S4 [file mbio.02711-22-s0004.tif]

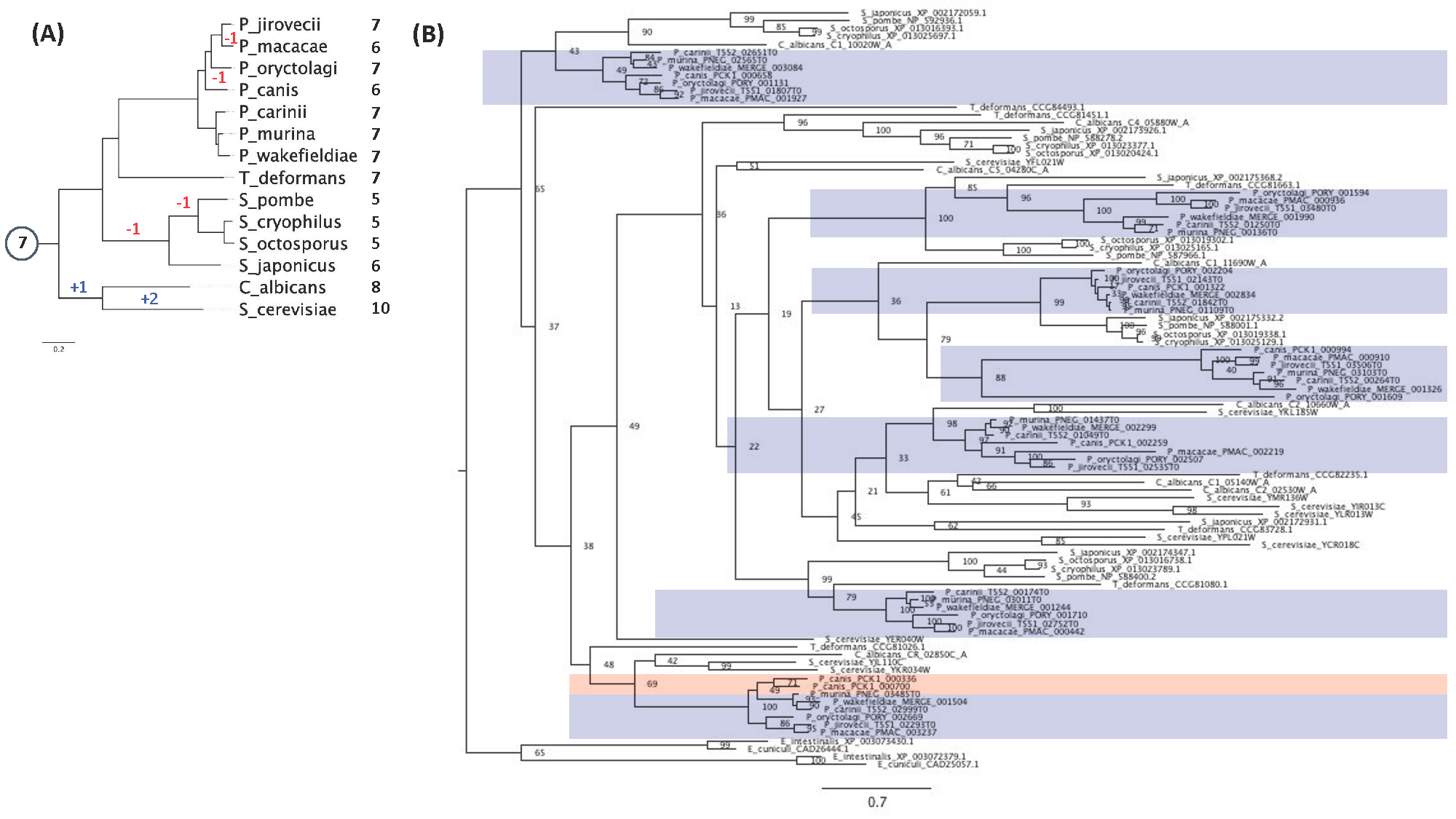

Supplement: FIG S5 [file mbio.02711-22-s0005.tif]

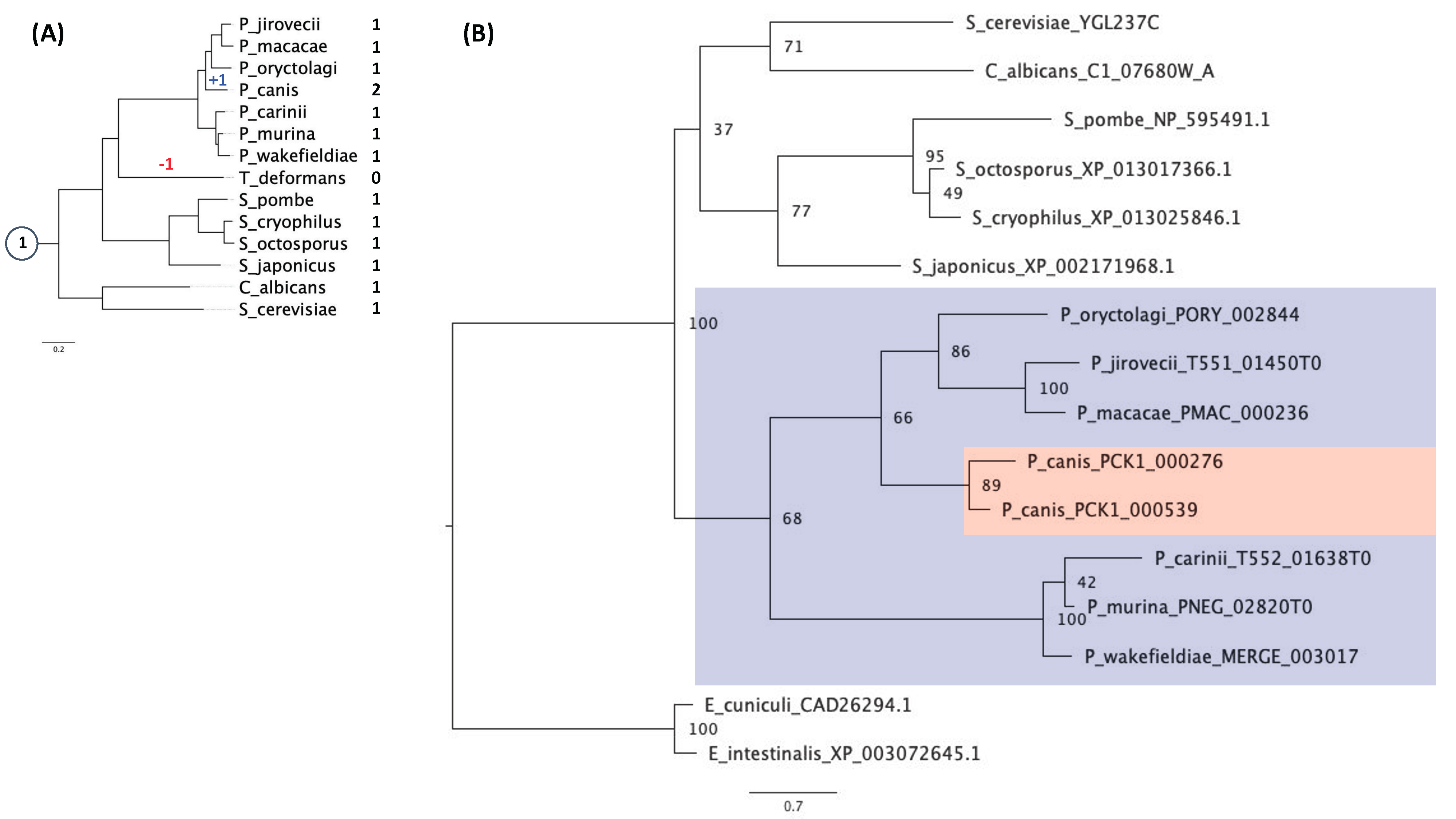

Supplement: FIG S6 [file mbio.02711-22-s0006.tif]

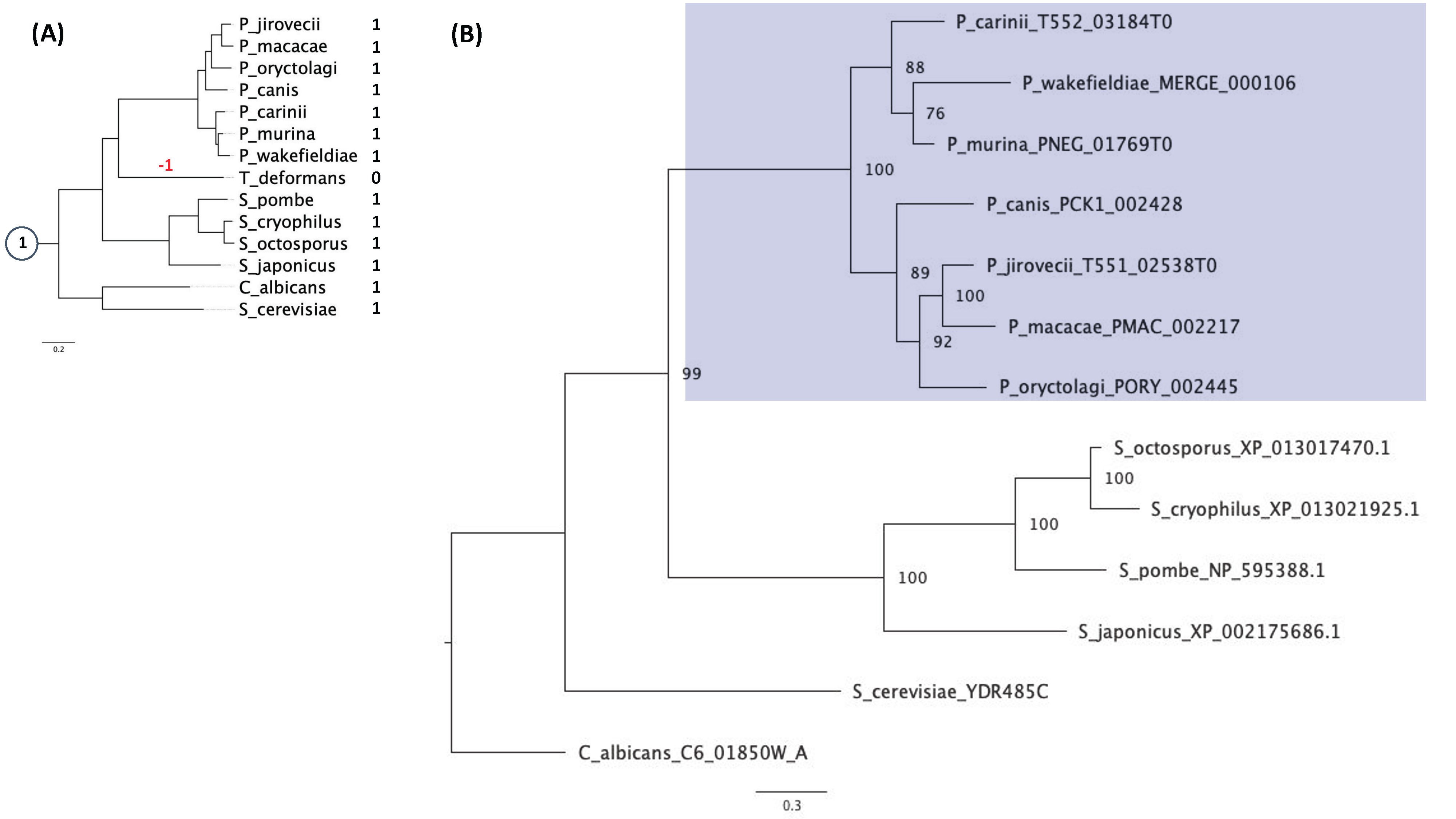

Supplement: FIG S7 [file mbio.02711-22-s0007.tif]

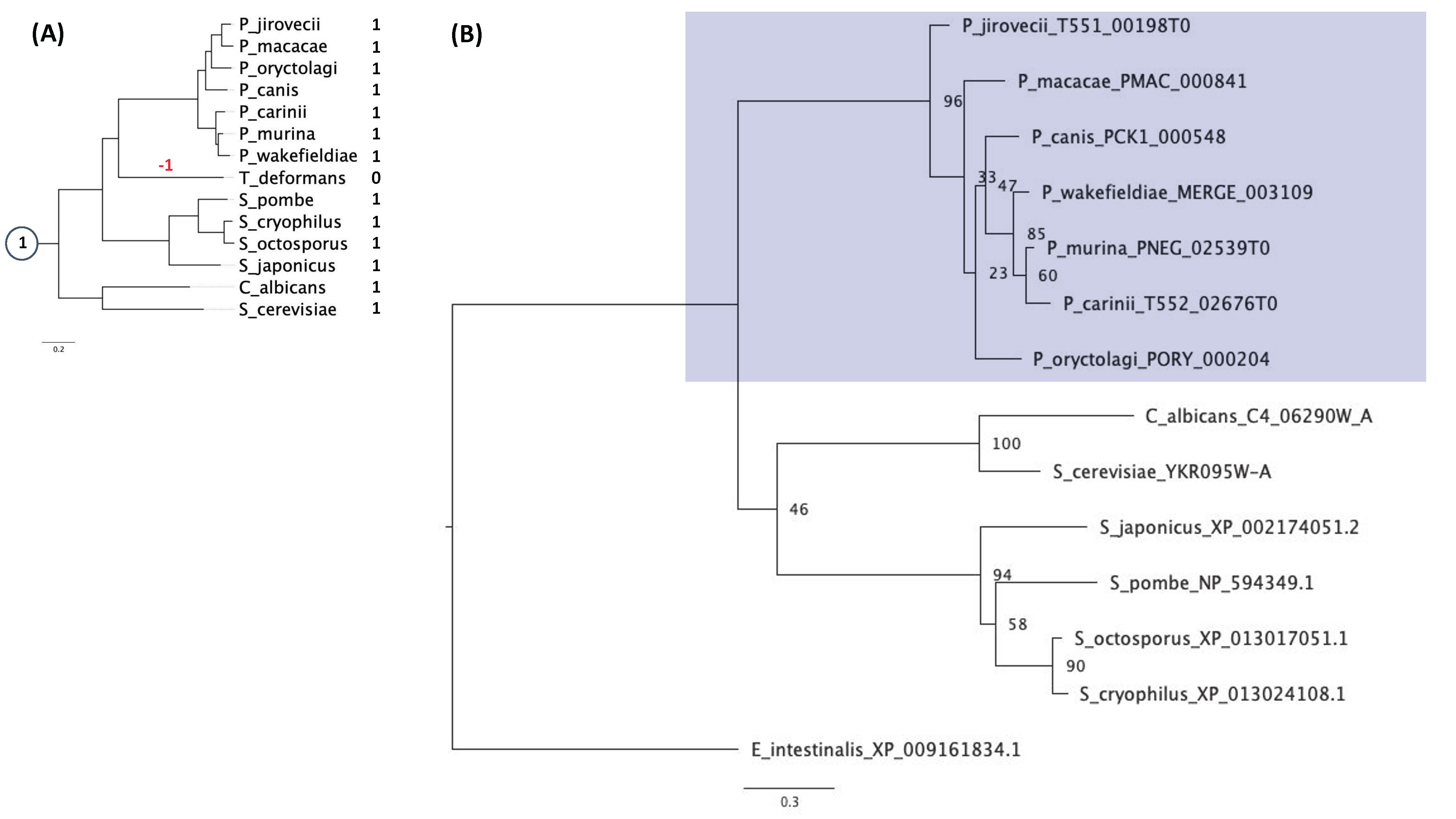

Supplement: FIG S8 [file mbio.02711-22-s0008.tif]

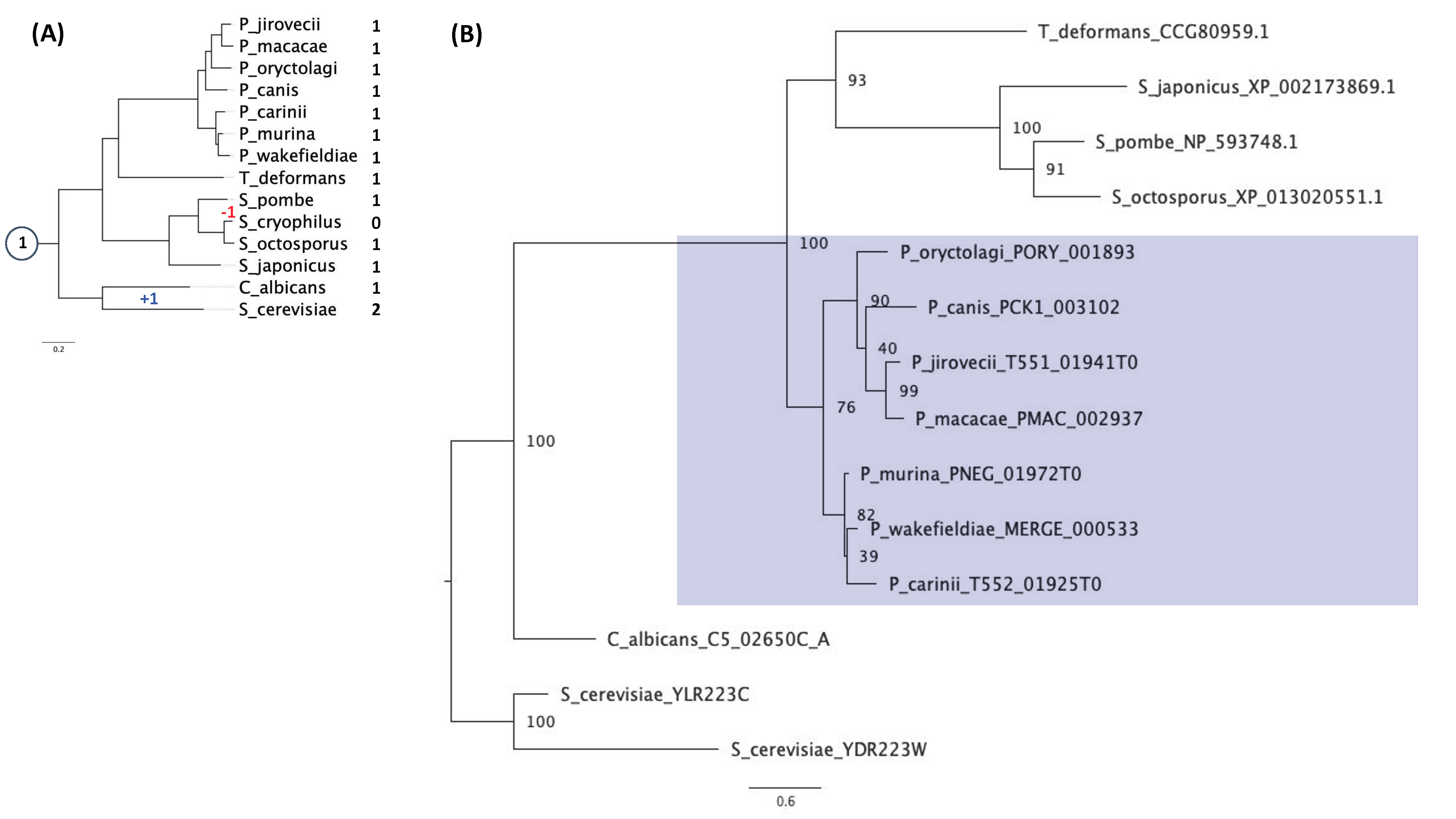

Supplement: FIG S9 [file mbio.02711-22-s0009.tif]
