# Supplementary material for: Analysis of Pneumocystis Transcription Factor Evolution and Implications for Biology and Lifestyle
Source: mBio. 2023 Jan 18;14(1):e02711-22. doi: 10.1128/mbio.02711-22 (PMC9973273; doi:10.1128/mbio.02711-22)
Supplement: TABLE S1 [file mbio.02711-22-s0010.docx]

**Table S1.** Species and strains used in this study together with their BioProject accession IDs and main characteristics of the genome assemblies.

| **Species** | **Strain/**  **isolate** | **BioProject accession ID** | **Genome size** | **Number of contigs** | **Contig N50** | **Annotated genes** |
| --- | --- | --- | --- | --- | --- | --- |
| *P. jirovecii* | RU7 | PRJNA223510 | 8.4 Mb | 70 | 454.6 kb | 3,811 |
| *P. murina* | B123 | PRJNA70803 | 7.5 Mb | 20 | 492.8 kb | 3,675 |
| *P. carinii* | B80 | PRJNA223511 | 7.7 Mb | 62 | 465.1 kb | 3,695 |
| *P. macacae* | P2C | PRJNA632025 | 8.3 Mb | 20 | 505.4 kb | 3,427 |
| *P. canis* | CK1 | PRJNA632556 | 7.9 Mb | 185 | 397.7 kb | 3,203 |
| *P. oryctolagi* | RABM | PRJNA632560 | 7.6 Mb | 273 | 372.8 kb | 2,856 |
| *P. wakefieldiae* | 2A | PRJNA632570 | 7.3 Mb | 17 | 480.7 kb | 3,223 |
| *S. cerevisiae* | S288C | PRJNA43747 | 12.1 Mb | 16 | 924.4 kb | 6,464 |
| *C. albicans* | SC5314 | PRJNA10701 | 14.3 Mb | 88 | 334.3 kb | 6,263 |
| *E. cuniculi* | GB-M1 | PRJNA13833 | 2.5 Mb | 12 | 218.3 kb | 2,029 |
| *E.* *intestinalis* | ATCC 50506 | PRJNA42703 | 2.2 Mb | 12 | 196.6 kb | 2,010 |
| *S. cryophilus* | OY26 | PRJNA38373 | 11.6 Mb | 198 | 290.1 kb | 5,494 |
| *S. japonicus* | yFS275 | PRJNA13640 | 11.7 Mb | 87 | 1.8 Mb | 5,224 |
| *S. octosporus* | yFS286 | PRJNA13639 | 11.6 Mb | 18 | 1.8 Mb | 5,347 |
| *S. pombe* | 972h- | PRJNA13836 | 12.6 Mb | 7 | 2.9 Mb | 6,974 |
| *T. deformans* | PYCC 5710 | PRJEA74523 | 13.4 Mb | 508 | 53.6 kb | 4,657 |
